# Critical Review of Biodegradable and Bioactive Polymer Composites for Bone Tissue Engineering and Drug Delivery Applications

**DOI:** 10.3390/polym13162623

**Published:** 2021-08-06

**Authors:** Shubham Sharma, P. Sudhakara, Jujhar Singh, R. A. Ilyas, M. R. M. Asyraf, M. R. Razman

**Affiliations:** 1 Regional Centre for Extension and Development, CSIR-Central Leather Research Institute, Leather Complex, Kapurthala Road, Jalandhar 144021, India; 2 PhD Research Scholar, IK Gujral Punjab Technical University, Jalandhar-Kapurthala, Highway, VPO, Ibban 144603, India; 3 IK Gujral Punjab Technical University, Jalandhar-Kapurthala, Highway, VPO, Ibban 144603, India; jujharsingh2085@gmail.com; 4 School of Chemical and Energy Engineering, Faculty of Engineering, Universiti Teknologi Malaysia, Johor Bahru 81310, Malaysia; ahmadilyas@utm.my; 5 Centre for Advanced Composite Materials, Universiti Teknologi Malaysia, Johor Bahru 81310, Malaysia; 6 Department of Aerospace Engineering, Faculty of Engineering, Universiti Putra Malaysia (UPM), Serdang 43400, Malaysia; 7 Research Centre for Sustainability Science and Governance (SGK), Institute for Environment and Development (LESTARI), Universiti Kebangsaan Malaysia (UKM), Bangi 43600, Malaysia

**Keywords:** drug delivery, biodegradable polymers, polymeric scaffolds, natural bioactive polymers, antimicrobial properties, anticancer activity, tissue engineering

## Abstract

In the determination of the bioavailability of drugs administered orally, the drugs’ solubility and permeability play a crucial role. For absorption of drug molecules and production of a pharmacological response, solubility is an important parameter that defines the concentration of the drug in systemic circulation. It is a challenging task to improve the oral bioavailability of drugs that have poor water solubility. Most drug molecules are either poorly soluble or insoluble in aqueous environments. Polymer nanocomposites are combinations of two or more different materials that possess unique characteristics and are fused together with sufficient energy in such a manner that the resultant material will have the best properties of both materials. These polymeric materials (biodegradable and other naturally bioactive polymers) are comprised of nanosized particles in a composition of other materials. A systematic search was carried out on Web of Science and SCOPUS using different keywords, and 485 records were found. After the screening and eligibility process, 88 journal articles were found to be eligible, and hence selected to be reviewed and analyzed. Biocompatible and biodegradable materials have emerged in the manufacture of therapeutic and pharmacologic devices, such as impermanent implantation and 3D scaffolds for tissue regeneration and biomedical applications. Substantial effort has been made in the usage of bio-based polymers for potential pharmacologic and biomedical purposes, including targeted deliveries and drug carriers for regulated drug release. These implementations necessitate unique physicochemical and pharmacokinetic, microbiological, metabolic, and degradation characteristics of the materials in order to provide prolific therapeutic treatments. As a result, a broadly diverse spectrum of natural or artificially synthesized polymers capable of enzymatic hydrolysis, hydrolyzing, or enzyme decomposition are being explored for biomedical purposes. This summary examines the contemporary status of biodegradable naturally and synthetically derived polymers for biomedical fields, such as tissue engineering, regenerative medicine, bioengineering, targeted drug discovery and delivery, implantation, and wound repair and healing. This review presents an insight into a number of the commonly used tissue engineering applications, including drug delivery carrier systems, demonstrated in the recent findings. Due to the inherent remarkable properties of biodegradable and bioactive polymers, such as their antimicrobial, antitumor, anti-inflammatory, and anticancer activities, certain materials have gained significant interest in recent years. These systems are also actively being researched to improve therapeutic activity and mitigate adverse consequences. In this article, we also present the main drug delivery systems reported in the literature and the main methods available to impregnate the polymeric scaffolds with drugs, their properties, and their respective benefits for tissue engineering.

## 1. Introduction

Bone tissue reconstruction represents one of the biggest challenges for medicine due to the existence of serious global health problems, such as diseases, defects, trauma, the rise of obesity, and sedentary lifestyles [1,2,3,4]. Bone tissue engineering is a recent field of research associated with regenerative medicine, and applies the principles of engineering and the life sciences toward the development of biological substitutes that restore, maintain, or improve tissue function [5,6,7]. Until recently, bone tissue reconstruction was represented by bone grafts, which present several limitations, such as disease transfer and cost. At present, a new generation of development is required in medicine that comprises not only physical support for bone formation, but also the presence of biochemical agents to promote the formation of the bone. One of the biggest advantages of this system is the fact that it enables controlled delivery of the drugs to the affected tissue [1,8,9].

To date, numerous porous nanocomposite scaffold materials have been investigated. However, these materials still present challenges due to their capability for regeneration and remodeling, and for mimicking the complicated physiochemical attributes of bone. In addition, the functionality of the scaffolds has been studied by loading biomolecules (drugs, growth factors (GFs)) onto the scaffolds to treat bone disorders or to act on the surrounding tissues [10,11,12].

**Figure 1:**
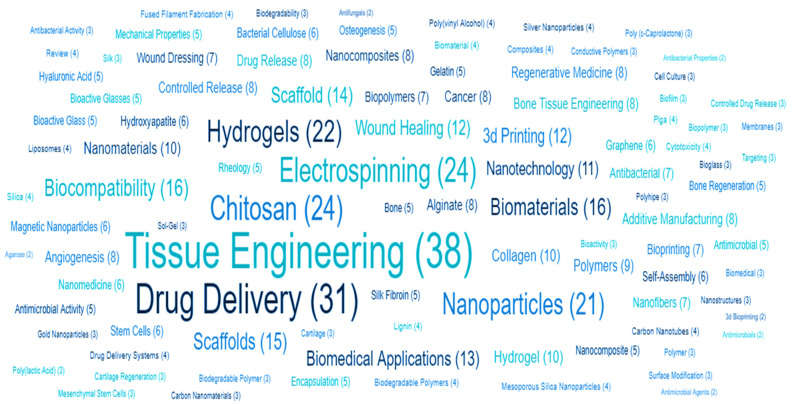
Infographic word-cloud visualization of the semantic clustering network of the keywords for physicomechanical, thermostability, and in vitro drug release studies of bioactive/biodegradable polymeric materials with remarkable biocompatibility in biomedical research.

Three-dimensional bone bioactive nanocomposite scaffolds can be fabricated from a wide variety of bulk biomaterials, such as bioceramic tricalciumphosphate (TCP), hydroxyapatite (HA), and bioglass (BG); or biodegradable polymer—collagen, chitosan, alginate, fibrin, polyesters, and polyethylene glycol (PEG) [13,14,15,16,17,18]. It was demonstrated that their composites represent a suitable alternative because they combine the advantages of both bioactive ceramics and biodegradable polymers for bone tissue engineering. The reason for this is simple: ceramics present weak mechanical properties due to brittleness (hard material with small elongation to failure) and the polymers present a deficiency in their compressive modulus compared with native bone tissue (polymers are typically too soft) [8]. Thus, these systems can reduce the disadvantages and offer new advantages in the case of bone tissue reconstruction. The Word-Cloud info-graphic plugin’s aim should be to provide succinct visually graphics representations of these kind of contextual features for better accessibility throughout intrusion-network mapping analysis of the existing-review as exhibits in the Figure 1. A word-cloud visualization of the keywords examined in this article is shown in Figure 1.

In tissue regeneration, the use of individual component scaffolds is widespread. Nevertheless, in certain circumstances, a single polymer is not able to fulfil all of the necessary criteria in several tissue regeneration applications. The bone matrix is a collagenous and apatite-based organic or inorganic composite. In addition to bone-tissue engineering, biocompatible composite scaffolds with an apatite element have been formed [19,20]. The most prevalently utilized material is perhaps hydroxy-apatite (HAP), which vaguely resembles the natural ingredients of bone. In addition, calcium phosphate (CaP) variants and bioglass have also been employed due to their excellent biocompatibility [21,22]. For instance, a PLGA/HAP-based nanofibrous composite scaffold was previously developed by polymer coating hydroxyapatite on to the PLGA scaffolds using a variety of methods [23,24]. Hydroxyapatite in composite scaffolds dramatically enhances proteinous adsorption capabilities, represses apoptosis cell death, and tends to create a more desirable microenvironment for bone tissue regeneration [25]. Nanohydroxyapatite polymeric composite scaffolds have been produced to imitate the nanosized characteristics of an organic mineral, in addition to emulating the inorganic–organic essence of natural bone [26,27,28]. Numerous inorganic elements have also been used to develop biotic and abiotic composite scaffolds. Lei et al. [29], for instance, utilized the temperature-dependent mediated phase-separation (TMPS) method to produce nanofibrous gelatinous silica hybrid scaffolds.

Due to their ability to mimic both the nanometer-scale architectural design and chemical content of innate organic bone extracellular matrices, mineralized nanofibrous scaffolds have been recommended due to their potential for scaffold material restoration. Macroporous nanofiber scaffolds for bone-like apatite deposition have been developed by incubating them in simulated body fluid (SBF) with an ionic electrolyte concentration and a pH closely comparable to that of human blood plasma [30,31]. After an acceptable period of incubation in SBF, it was found that CaP could be deposited immediately upon the substratum, and a homogeneous, fibrous, compact and dense layer of nano-apatite was developed to portray an intrinsic interlayer of a porous wall surface without clogging the macropores [32,33,34,35,36].

**Figure 2:**
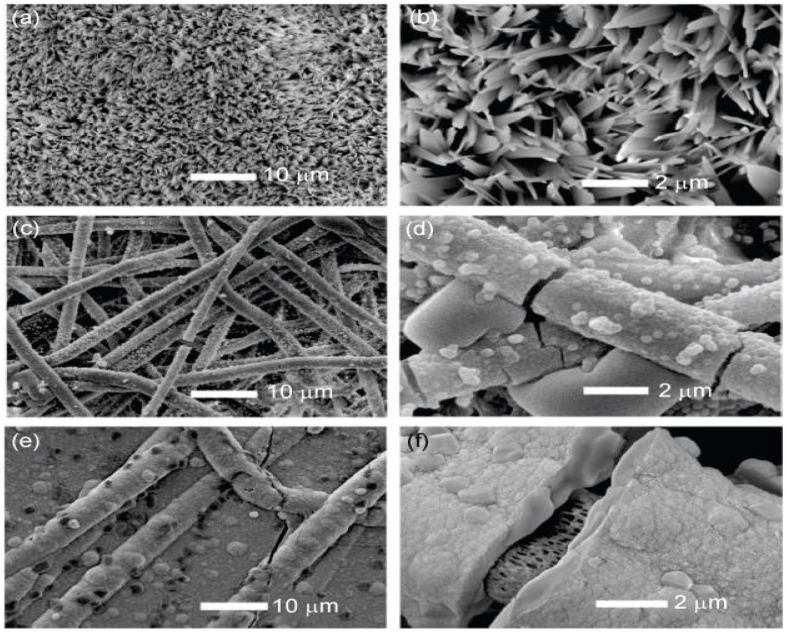
Microstructure of mineralized PLLA matrices: (**a**) electro-deposition at 3 V, 60 °C for 1 h; (**b**) high-magnification picture of (**a**,**c**) mineralized in 1.5 SBF for 12 days; (**d**) magnified image of (**c**,**e**) mineralized in 1.5 SBF for one month; and (**f**) magnified picture of (**e**). Reproduced with permission from [33].

An electro-deposition methodology was previously proposed to reduce the mineralization period to below 1 hour [32]. In terms of the dissolution rate, the chemical composition, and the microstructure of CaP developed on electrospinning-based PLLA-fiber skinny-matrices, a previous study [33] recently contrasted a novel electro-deposition technique with a well-known SBF incubation approach. Based on the mineralization of a fibrillar matrix, electro-deposition appears to have been two–three orders of magnitude quicker than the SBF technique. The aim of the study was to reduce the mineralization period from fourteen days to one hour while achieving the same degree of mineralization (Figure 2). Steadily increasing the fiber diameter led to faster mineral deposition in the electro-deposition technique compared to the relatively slower mineralization of the SBF incubation approach. The chemical structure and morphological characteristics of CaP can be obtained by altering the electric-deposition prospective and electrolyte temperatures to tune the blend of brushite and hydroxyapatite. The SBF technique can only generate a minimal HAP. Mineralized electrospinning-based PLLA fibrous scaffolds acquired by either technique strengthen the proliferative and osteogenesis differentiation of pre-osteoblastic MC3T3-E1 cells to a level comparable with that of the neat PLLA matrix.

**Figure 3:**
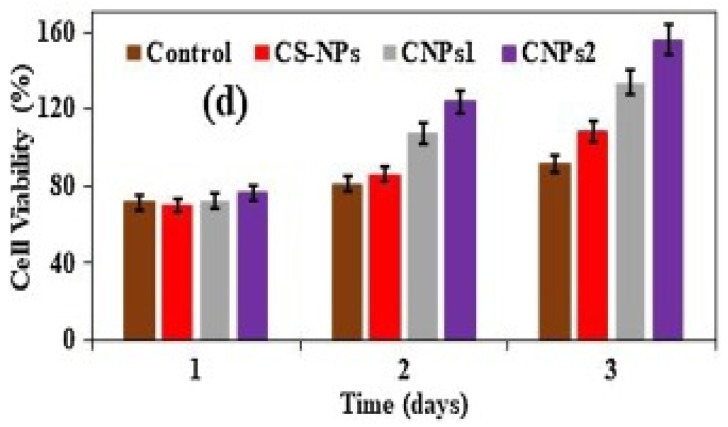
Cell viability of chitosan (CS) nanocomposite hydrogel nanoparticles (CNPs). Reproduced with permission from [34].

Mamidi and Delgadillo [34] adopted an ionic gelation strategy to develop chitosan (CS) nanocomposite hydrogel nanoparticles (CNPs). The authors observed disassembling of the CNPs’ structure at 55 °C. In addition, the CNPs showed good cell viability against human fibroblast cells, as exhibited in Figure 3. 

**Figure 4:**
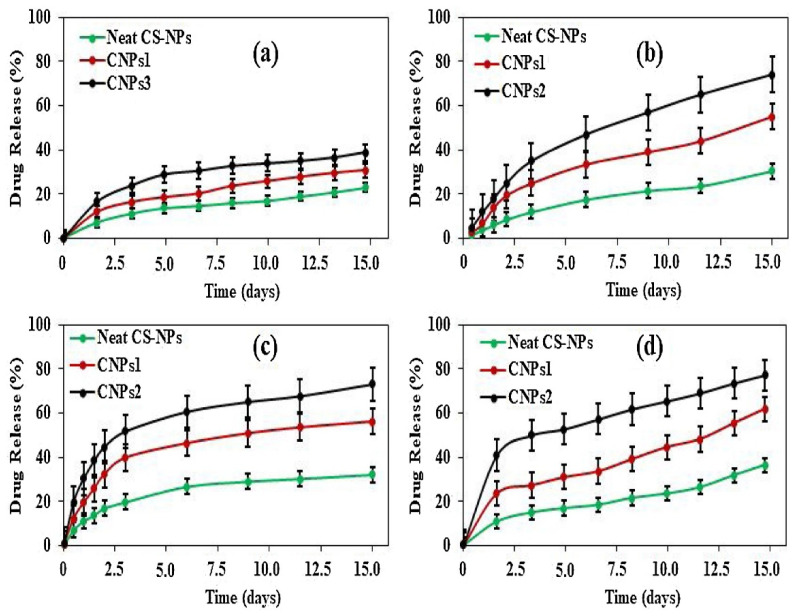
Diclofenac-embedded pure CS-NPs, CNPs1, and CNPs2 in vitro release of drugs studied at 37 °C in varying acid levels and environmental conditions: (**a**) pH 2, (**b**) pH 6, (**c**) pH 7.4, and (**d**) pH 9. The results are presented as an average deviation of ±3. Reproduced with permission from [34].

Therefore, CNPs provide a better pH and temperature-triggered drug delivery platform for the GI tract and colon-targeted drug delivery, in addition to the highest drug release under specific pH and temperature values, as displayed in Figure 4.

**Figure 5:**
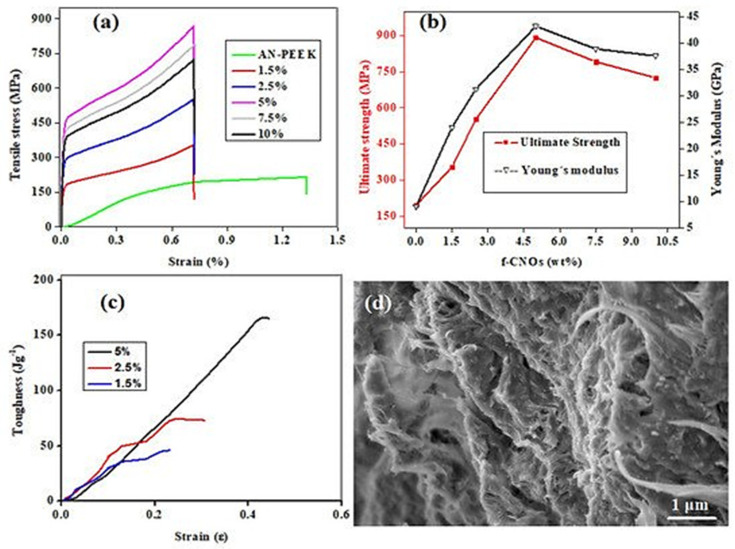
Physicomechanical analysis of biopolymer composite films employing quantitative techniques: (**a**) load displacement; (**b**) ultimate strength elastic moduli; and (**c**) impact–strength–strain graphs from uni-axial tensile nanocomposite thin films. (**d**) SEM micrographs of the fractography of biopolymeric nanocomposites comprising 5% of f-CNOs, produced by tear testing on perforated grooved specimens. Reproduced with permission from [35].

In another study, Mamidi et al. [35] developed PAPMA-CNOs and AN-PEEK biopolymer matrix composites. The authors observed that interactions between CNO and DOX played a vital role in controlling the drug release from the thin film. This film possessed a tensile strength of 891 MPa, an elastic modulus of 43.2 GPa, and a toughness value of 164.5 J/g. This thin film is used in numerous medical applications. To examine the fracture deformation phenomenon during the tensile test, the researchers analyzed the delamination zone under tensile loading. The sheets had a relatively uniform homogeneous and compact morphology, as illustrated in Figure 5.

Polymeric materials, among other materials, have been formed as tissue engineering scaffolds. These materials have relatively higher processing and handling versatility, processability, adaptability, degradability, and biocompatibility, which can be augmented via structural design analysis [36]. Polymeric materials (such as natural polymers, natural polymeric-derived composites, and synthetic polymeric materials, in addition to synthetic polymeric materials made of natural monomeric units and amended to natural moieties) are therefore the primary scaffold materials used in tissue engineering [37]. This descriptive analysis summary is not intended to be a rigorous exhaustive analysis of all of the polymeric materials used in biomedical applications, bioengineering, regenerative medicine, bone regeneration, or tissue regeneration. 

### 1.1. Advances in Biodegradable Polymers for Biomedical Applications

The biodegradable polymers can be classified as natural polymers or synthetic polymers based on their origin. Natural polymers are further classified as polysaccharides, polypeptides, and polyesters, depending on their repeating units. The polysaccharide contains d-glucopyranoside repeating units, e.g., starch, cellulose, chitin, and chitosan. Naturally occurring polypeptides contain amino acid repeating units. Among the naturally occurring polypeptides, gelatin is extensively used in pharmaceutical and biomedical applications. Naturally occurring polyesters, for example, polyhydroxybutyrate (PHB) and polyhydroxyvalerate (PHV), are potential candidates for biomedical applications [38]. Natural materials are generally biocompatible, and exhibit mechanical properties comparable to those of native tissues. However, these materials also suffer from disadvantages, such as limited control over physicochemical properties and difficulties in modifying degradation rates. Purification and sterilization of these biomaterials after isolation from different sources is relatively cumbersome [39].

In oral drug delivery, cellulose derivatives, for example, cellulose ethers such as ethyl cellulose, methyl cellulose, hydroxypropyl methylcellulose (HPMC), and hydroxypropyl cellulose, have been used in the form of coatings. Similarly, synthetic polymers such as poly(acrylates), poly(methacrylates), poly(methyl methacrylates), poly(hydroxyethyl methacrylates), and copolymers thereof have been extensively used [40].

Stimuli-sensitive polymers are another class of polymers that interact and respond to the environmental conditions, such as temperature, light, salt concentration, and pH [41]. pH-sensitive polymers are used to develop smart delivery systems due to the variation in the physiological pH in different parts of the body. However, although these polymers are biocompatible, they are not biodegradable. The use of polymeric carriers in injectable drug delivery systems requires biodegradable polymers that degrade into nontoxic and safe products.

PLGA and PLLA are used to encapsulate drugs in the form of microparticles or nanoparticles to increase the circulation time and the bioavailability of the drug [42]. New drug delivery systems have been developed for chronic diseases and/or conditions that require sustained drug delivery.

The sustained release of drugs was achieved initially by drug diffusion from polymeric microspheres followed by polymer degradation [43].

The development of injectable in situ semisolid drug depots has been explored as an alternative delivery system. Biodegradable polymers can be used in the form of an injectable matrix or a depot for drug delivery, and as injectable scaffolds in tissue engineering [44]. Atrix laboratories developed ATRIGEL^®^ technology, in which sustained release of Leuprorelin acetate was achieved through a PLGA depot formed in situ.

The literature indicates that polymer-based scaffolds have been developed that can be used in tissue engineering as supports for cell attachment and proliferation [45,46,47,48,49,50]. These biodegradable scaffolds can be simultaneously used as cell support and for controlled delivery of biologically active proteins, such as growth factors and cytokines [51]. In addition to biodegradability, the polymer should satisfy the following requirements for processability:

- It must be liquid so that it can appropriately fill the cavities and replicate the patterns present on mold with high fidelity.
- It must contain functional groups to enable cross-linking during processing.

PEG and PLA block macromers containing terminal (meth) acrylate groups were photopolymerized to yield highly cross-linked biodegradable materials [52]. In another effort to incorporate vinyl functionality in biodegradable aliphatic polyesters, di- and triblock copolymers of ε-caprolactone (CL), glycolide (GA), and lactide (LA) with ethylene oxide (EO) bearing terminal vinyl groups were developed. These polymers were further processed by UV-micro-embossing to fabricate biodegradable scaffolds. However, these polymers must be processed at 65 °C because they are not liquids at room temperature [53,54].

Photo-patternable biodegradable 2-hydroxylethyl methacrylate (HEMA) conjugated poly(*ε*-caprolactone-*Co*-RS-*β*-malic-acid) (PCLMAc) copolymers were synthesized to fabricate biodegradable scaffolds. However quantitative conjugation of carboxylic acid groups to HEMA ester is difficult and its synthesis involves multiple steps. Furthermore, the resulting polymers are not liquids at room temperature and need to be processed at 60 °C [55].

Liquid photo-patternable polyurethane diacrylates were developed to fabricate biocompatible scaffolds by UV-micro-embossing. The fabricated scaffolds exhibited cytotoxicity due to the presence of unreacted monomers and the photo initiator used during polymer synthesis. The polymers were rendered biocompatible after the residues were leached out by repeated extractions with methanol, which is not a highly desirable solvent [56]. Other examples of photopolymerizable and degradable polymers developed to date include poly(propylene fumarate) (PPF), photo-cross-linkable poly(anhydride), polyethylene glycol, and cross-linkable poly(saccharide). However, synthesis of these polymers involves multiple reactions and purification steps [57].

Thus, biodegradable polymers are extensively used in numerous biomedical applications, including as drug carriers, tissue regeneration, regenerative medication, gene therapy, temporary implantable devices, and coatings on implants. The basic criteria for choosing a polymer to be used as a degradable biomaterial are: (i) the mechanical properties and degradation rate should match the needs of an application so that adequate strength remains until the surrounding tissue has been cured; (ii) biocompatibility; (iii) non-toxic degradation products; (iv) shelf life or stability; (v) processability and cost. For drug delivery applications, the time of release governs the type of polymer, and the shape and size of the device. For example, lactide and glycolide polymers are clinically approved polymers that can be used in any application [58,59]. 

Biodegradable plastics are also widely used in agricultural areas. The main motive for using biopolymers in agricultural sectors is the rising utilization of polymers in agriculture, which has enabled farmers to enhance their crop production. Some of the plastics employed in agriculture are recyclable, such as silage films, greenhouse sheet layers, fertilizer satchels, tubing pipelines, and other polymer materials, whereas others are difficult to recycle, such as thin mulching films, thin low tunnel films, and direct covering films. These covering layer sheets are very slender and flimsy, and are often heavily degraded with dust, dirt, and unwanted substances. Thus, an attractive alternative for nonrecyclable plastic waste is biodegradation. Consequently, the utilization of biodegradable plastics is increasing in agricultural applications. These applications primarily include mulching films, plant pots, and compost bags [60,61]. 

Although biodegradable polymers can be used in different applications, such as the packaging, medical, and agricultural fields, the commercialization of biodegradable polymers is often hampered due to competition with commodity plastics, which are cheap and familiar to the customer. In addition, the infrastructure needs to be developed for the disposal of biodegradable polymers in bioactive environments, which requires capital investment [62]. Moreover, the biodegradable polymers currently available possess inferior physical properties, such as poor strength and dimensional stability, and their processing is technically difficult [63]. 

Synthetic biodegradable polymerics are regarded as being biodegradable, biocompatible, and highly safe. As a result, they are widely used in biomedical applications, and particularly in the areas of controlled drug carrier systems and tissue regeneration. Due to the degradable nature of polymeric implants, there is no requirement of surgical intervention to remove the implant at the end of its functional life [64]. In tissue engineering applications, synthetic polymers are mainly used in scaffolds, which provide suitable mechanical support and show favorable surface properties, such as adhesion, proliferation, and differentiation of cells [65]. 

Polylactic acid is produced either through the fermentation process of carbohydrate crops (such as corn, sugar beets, tapioca roots, wheat, barley, and sugarcane) or chemical synthesis [66]. The fermentation process is preferred over the synthetic route because the latter is unable to produce the desirable l-isomer, in addition to its high manufacturing cost. In contrast, the fermentation process produces the l-isomer with a high purity (99.5%). In general, PLA is produced from the pure l-isomer.

PLA is principally produced via different processes: condensation polymerization of lactic acid (LA), condensation reaction in an azeotropic solution, and ring opening polymerization of an intermediate called lactide. The first method (polycondensation) involves the esterification of monomers in the presence of suitable solvents, and water (byproduct) is removed azeotropically under reduced pressure (vacuum) and high temperature. Tin (II) chloride is the most commonly used catalyst in this method and can be recovered at the end of the reaction. This method is the least expensive route but cannot produce solvent-free high molecular weight PLA having superior mechanical properties. The second method involves the condensation reaction of lactic acid in an azeotropic solution. The third method appears to be the most commonly employed procedure for producing higher molecular weight PLA. This approach involves three steps: (i) condensation of lactic acid monomeric subunits; (ii) depolymerization of the PLA to the lactic acid; and (iii) the cyclization ring opening polymerization of the lactide unit in the presence of metal catalysts, resulting in PLA with a high molecular weight [67].

Furthermore, PLA exists in three stereoforms: PLLA, PDLA, and PDLLA. Of these, PLLA and PDLA are semicrystalline polymers that show a high tensile strength and low elongation, whereas PDLLA is a more amorphous polymer and shows a random distribution of both of the isomers [68]. The ROP of the l-lactide unit can be performed via melting or a suspension solution using stannous octoate (SnOct2) as the initiator, which avoids racemization at high temperature and transesterification.

Polylactic acid has a vast range of applications. However, it cannot be used in flexible films due to poor ductility, and poor thermal and barrier properties [69]. PLA is also extensively used in medical applications due to its unique characteristics, such as biodegradability, biocompatibility, ecofriendliness, and thermoplastic processability. Moreover, PLA works very well and offers outstanding properties at a low price. It is used for preparing various devices, such as degradable sutures, nanoparticles, and drug-releasing microparticles. The use of biodegradable polymers rather than nondegradable polymers in medical applications has the advantage that it eliminates the need to remove implants; the biodegradable polymers remain temporarily in the body and disappear on degradation [70]. The physical properties of PLA (such as transparency or the mechanical properties) are comparable to those of polystyrene and poly(ethylene-terephthalate), but due to its high cost (compared to PP, PE, PS, etc.), brittleness, low viscosity, medium gas barrier properties, and high moisture sensitivity, its use is restricted to specific applications. Thus, efforts are being made to improve the properties of PLA by blending. Investigations have been conducted on blends of PLA with other polymers, such as poly(ε-caprolactone), poly(hydroxyl butyrate), polyethylene glycol, and poly(hexamethylene succinate). However, the produced blends were immiscible and resulted in poor mechanical properties [71,72,73].

Polycaprolactone (PCL) is a synthetic linear polyester derived from crude oil. It is resistant to water, oil, solvents, and chlorine. The average molecular weight of PCL ranges from 3000 to 90,000 g/mol. With an increase in molecular weight, the crystallinity of PCL tends to decrease. It is fully biodegradable under composting conditions and mainly used in the biomedical field. It acts as a stiffening material for shoes and orthopedic splints, and in completely biodegradable compostable bags, fibers, and sutures. It is also used in thermoplastic polyurethanes, adhesives, resins, etc. [74,75]. PCL is also used in tissue engineering [76].

PCL is readily biodegradable in diverse environments, such as marine water, soil, sewage sludge, and compost ecosystems; hence, it is widely used in drug delivery systems. The biodegradation of PCL occurs through either enzymes, simple hydrolysis, or both.

There are several parameters that influence the biodegradation of PCL, such as the molecular weight, crystallinity, thickness of the films, and degradation parameters. The microorganisms secrete extracellular depolymerases that degrade the polymer.

The enzymatic degradation of polycaprolactone has been studied mainly in the presence of lipase enzymes, which help in accelerating the biodegradation of PCL [77], for example, Rhizopus delemer lipase [78], Rhizopus arrhizus lipase [79], and Pseudomonas lipase [80,81]. 

Studies have been performed on the biodegradation of PCL [82]. Chen et al. (2000) observed that the enzyme lipase can accelerate the degradation of polycaprolactone microparticles, and the degradation rate of PCL is not significantly influenced by its surface area [83]. Murphy et al. (1996) revealed that the depolymerized enzyme produced by *Fusarium moniliforme* is cutinase [84]. Oda et al. (1995) isolated five fungal strains having the capability to degrade two polymers: PHB and PCL [85]. One of the fungal strains was identified as Paecilomyces lilacinus. The degradation of polycaprolactone was also studied using the bacteria Alcaligenes faecalis [82,85,86]. Abdel-Motaal et al. (2014) found that Pseudomonas japonica-Y7-09 (yeast) produced the extracellular enzyme cutinase, which degraded PCL by 93.33% in 15 days [87]. The mechanism of biodegradation of PCL has also been studied in detail. It is believed that PCL depolymerases preferentially attack the amorphous areas of polymers and degradation occurs due to endo- and exo-cleavage [82].

The various physicochemical and physicomechanical characteristics of polycaprolactone appear to be modified by either copolymerization or by efficiently blending with other polymers. The copolymerization helps to alter the chemical property of PCL, which further affects numerous properties, such as crystallinity and solubility, resulting in a modified polymer that has the desired attributes for drug delivery. By comparison, blending helps to change the physical properties and biodegradation, in addition to the mechanical properties, resulting in polymers that are preferred for tissue engineering. PCL has been found to be compatible with natural polymers (starch, hydroxyl apatite, and chitosan), polyethylene oxide (PEO), and polylactic acid and polylactic co-glycolic acid (PLGA). These modifications are useful in formulations for drug delivery [88].

Polyglycolic acid is produced by the polycondensation reaction between glycol and aliphatic dicarboxylic acids. The constituents are derived from renewable resources, such as glycol obtained from glycerol, and organic acids are obtained via fermentation. PGA is a soft and biodegradable material, and possesses good sensitivity and a high melting point (approximately 200 °C). It has excellent material properties similar to those of aromatic PET. PGA is commercially produced by Dupont, either in the form of an aliphatic-aromatic copolymer (Biomax^®^) or as aramid fibers (Kevlar^®^) [89].

PGA and its copolymers are both widely utilized in medical applications for degradable and absorbable sutures. They can easily degrade in aqueous surroundings, such as body fluids, via hydrolysis of the ester backbone. Furthermore, the degraded products of PGA are metabolized to CO_2_ and water [90,91].

Microbial polymers/polyhydroxyalkanoates (PHAs) are biodegradable biopolyesters that are completely synthesized by microorganisms, such as bacteria and fungi, in addition to some plants. There are many bacteria that can synthesize PHA, such as those found in activated sludge, oceans, or extreme environments. More than 30% of soil-inhabiting bacteria are capable of synthesizing PHA [83,92]. Some examples of PHA-producing bacteria are Alcaligenes latus, Pseudomonas oleovorans, and Azotobacter vinelandii [93]. Microorganisms such as Ralstonia eutropha and recombinant Escherichia coli are capable of accumulating PHA in quantities of as much as 90% (*w*/*w*) of their dry cell mass in a nutrient-limited media, i.e., media that is deprived of essential nutrients such as nitrogen, phosphorus, or oxygen, but in which an excess of carbon is present. The most general limitation is observed with nitrogen (*Azotobacter* spp.), but the most efficient limitation is that of oxygen. Due to the insolubility of PHA in water, it accumulates as carbon or an energy source within the intracellular granules [83,92,94].

Poly3-hydroxy-butyrate (PHB) is the most common and well-studied polymer of the polyhydroxyalkanoate family. It has been reported that this bacterium can accumulate PHB intracellularly. It is a homopolymer made up of 3-hydroxybutyric (3HB) acid molecules. The molecules are joined by ester bonds formed between the 3-hydroxyl group of one monomer and the carboxylic group of another. Numerous other bacteria have been identified as accumulating PHB in their cells, both aerobically and anaerobically. However, PHB possesses poor physical properties, and is too stiff and brittle to be used in most commodity products. It was subsequently found that PHA in activated sludge contains monomers other than 3-hydroxybutyric acid (3HB), such as 3-hydroxyvalerate (3HV). The incorporation of a few percent of 3HV units in the polymer helps to improve the flexibility and also reduces the brittleness. Numerous companies, such as ICI and, subsequently, Zeneca and Monsanto, started production of PHBV at an industrial scale [95,96,97,98]. Their production capacity has increased to 900 million tons per year. The commercial PHA produced by Tianan contains about 5% ester of valeric acid, although some experimental grades contain up to 15% valerate. Valerate improves the flexibility of the polymer. 

PHAs are thermoplastic and/or elastomeric, biocompatible, non-toxic, enantiomerically pure, optically active (i.e., possess only the R-configuration), piezoelectric (i.e., assist in wound repair and healing), and also induce bone regeneration and formation. They show better resistance to UV degradation than polypropylene (PP) but are less solvent resistant. The most important characteristic is that they are completely biodegradable. Due to these properties, PHAs are widely used in biomedical applications, such as orthopedy (screws, bone graft substitutes, and scaffolds for cartilage engineering), cardiovascular system devices, wound management (sutures, dressings, and dusting powders), urological stents, and controlled drug delivery (tablets, micro-carriers, and implants). Like PVC and PET, PHAs also exhibit good barrier properties, so they are also used in packaging applications, such as shampoo bottles, cosmetic containers, milk cartons and films, cover for cardboard and paper, pens, combs, bullets, and moisture barriers in nappies and sanitary towels. PHA may help to address the problems of environmental pollution caused by nondegradable synthetic polymers [99,100,101,102].

There are some drawbacks of using these polymers: (i) the cost of producing PHAs is very high compared to conventional petroleum-based plastics; (ii) the processing of PHAs is more difficult than conventional petroleum-based plastics due to their slow crystallization process; (iii) their mechanical and thermal characteristics are not consistent compared to those of petrochemical plastics; (iv) they are further required to be developed for a wider range of applications and large-scale production; (v) the quality and uniformity of PHA must be optimized.

The two polyesters, namely, PLA and PHA, have their own advantages and disadvantages. Typically, polylactic acid (PLA) is cheaper than PHA. Therefore, the application research of PLA is more advanced than that of PHA [83,92].

Roy et al. (2008) examined the biodegradation of PE containing a pro-oxidant (cobalt stearate) using a consortium of three bacteria, namely, Bacillus cereus, Bacillus pumilus, and Bacillus halodenitrificans [103]. The films were UV irradiated (λmax at 313 nm) and subsequently incubated with the bacteria. The degradation was monitored based on the FTIR, mechanical properties, GC-MS, DSC, TGA, SEM, melt flow index, weight loss, and cfu count. It was observed that there was decrease in the carbonyl index (by FTIR analysis); the formation of low molecular weight compounds (by GC-MS studies); an increase in initial decomposition temperature (TGA); the formation of biofilm on the polymer surface (by SEM analysis); a weight loss of polymer of 8.4%; and an increase in the bacterial count (by cfu count).

Reddy et al. (2008) developed the blends of PLA/PP to create fibers and characterized them via their mechanical properties, and SEM, XRD, and DSC techniques [104]. The blends showed partial compatibility between PLA and PP, and their mechanical properties were inferior to those of the pure polymers. However, the blends showed better resistance to hydrolysis and biodegradation, in addition to better dyeability, than pure PLA. Nishida et al. (2009) prepared blends of PLLA and PP with and without the catalyst MgO, and characterized them using SEC, NMR, FTIR, SEM, and TGA techniques [105]. TGA analysis showed that the addition of MgO to the blend selectively accelerated the depolymerization of the PLLA component in the blend, leading to the generation of L, l-lactide as a main volatile product. Hamad et al. (2011b) blended PLA with PP in different ratios and studied their rheological and mechanical properties [106]. The rheological results of blends revealed that the true viscosity was between that of the pure polymers, whereas the flow activation energy was less than that of the pure polymers. The mechanical tests showed that there was incompatibility between the two polymers. Choudhary et al. (2011) blended PLA with PP in various ratios with and without compatibilizers, i.e., maleic anhydride grafted PP (MAPP) and glycidyl methacrylate [107]. The blends were characterized by mechanical tests, and DSC, TGA, FTIR and SEM techniques. The results revealed that a blend of PLA/PP in the ratio of 90:10 had optimum mechanical properties, which led to improved melt processability of PLA. The interaction between these two polymeric materials can be improved by the addition of a suitable compatibilizer, such as MAPP. MAPP is an effective compatibilizer that mediates the polarity at the interface of two polymers.

### 1.2. Developments in Bioactive/Biodegradable Polymers with Interfacial Activity-Assisted Surface Functionalization for Drug Delivery

Polymers are macromolecules formed from the combined repetitive monomer units. Many of these polymers excel in transporting the drug to the diseased site and releasing it in a controlled manner [108]. Their structures help protect the drug and thereby increase its bioavailability. Polymers are broadly classified as biodegradable and nonbiodegradable. The latter have some advantages and are also employed in drug delivery [109]. Occasionally, after the drug is released, nonbiodegradable polymers need to be retrieved using invasive methods. In contrast, biodegradable polymers do not require retrieval because they degrade/erode into smaller molecules that are eliminated through different metabolic pathways. 

Biopolymers are mainly classified as natural (chitosan and cellulose) and synthetic (PLGA, polyanhydrides), as illustrated in Table 1. Synthetic biopolymers have greater significance because they can be altered to suit specific requirements. The drug release rates can be controlled, and drugs of different physiochemical properties can be accommodated [110] without altering their therapeutic efficacy, thereby making them one of the most researched polymers in the field of drug delivery. Many biopolymers have transitioned from academic curiosities to real-world applications, and many water-soluble polymers (Table 2) play an essential role as drug delivery agents [111]. 

Nonbiodegradable polymers, by comparison, have been employed extensively in biomedical devices, such as catheters, heart valves, prostheses, and dialysis membranes, for a substantial period. All of these polymers are hydrophobic, and their usage in the field of drug delivery has been limited due to their degradability issues. Hence, they mainly act as reservoirs for the drugs, and need to be retrieved surgically or after depletion of the drug.

Synthetic degradable polymers, such as polyesters, mainly display bulk erosion [113]. The polymer matrix becomes porous as time progresses, leading to the release of the drug into its environment due to the matrix’s sudden collapse. Polymers, such as polyanhydrides, predominantly experience surface erosion as they undergo hydrolytic bond cleavage to form products that dissolve slowly in water [113,114]. Most of the biodegradable polymers display a combination of bulk and surface erosion, leading to varying drug release profiles. Polymers that predominantly degrade via bulk erosion initially show a first-order release, followed by a slow, constant release phase. Slower degradation of polymers such as PLGA is possible by employing higher molecular weight, leading to a slower, controlled drug release. Some of the well-known water-soluble polymers, such as polyethylene glycol (PEG), are either employed as standalone drug carriers [115] or conjugated to other polymers to provide properties of stealth [116]. They also help increase the stability of the drug.

Among all of these available prospects, PLGA, PEG, and PLA are a few of the FDA- approved polymers [117] and, thus, are most widely incorporated in the field of drug delivery.

For polymers in drug delivery, PLGA is the gold standard as a drug delivery vehicle. It is one of the most extensively employed biodegradable polymers for delivering drugs, proteins, DNA, and other bioactive agents [118]. PLGA is a copolymer produced by the combination of PLA and PGA, and is commercially available as 50:50, 65:35, and 75:25 PLGA, among other variants. The value 75 (in 75:25) denotes the percentage quantity of PLA, and the remainder (25) is that of PGA. Varying the concentration of PLA and PGA helps obtain the desired properties in the copolymer. The higher the concentration of PLA, the slower the degradation rate, with some exceptions [119]. The properties of the polymer can be tuned for the drug by varying the molecular weight, the ratio of PLA and PGA, the amount of the polymer, and the drug, to obtain the desired protection and release profile.

PLGA microparticles loaded with triptorelin were synthesized by Mahboubian et al. using the double emulsion solvent evaporation technique. The effect of various parameters, such as the emulsifying agent, volume of the water phase, and addition of NaCl, was studied [118,119].

Xie and colleagues formulated PLGA NPs to deliver Paclitaxel (PTX) [120]. To evaluate the formulation’s effectiveness in passing the blood–brain barrier (BBB) and attaining the desired tissue, they used MDCK/C6 cell lines and varied the additives and coatings. The uptake results indicated that PLGA NPs with additives showed a higher uptake than those with surface coatings. Cell viability was also low when the cells were treated with PLGA NPs compared to the control (no treatment) [120]. Similarly, Averineni et al. encapsulated the drug in PLGA NPs (50:50) and assessed its antitumor activity on BT-549 cell lines. The optimized formulations released the drug for 15 days and had an inhibitory effect for around 7 days with a lower clearance rate [121].

Sengel-Turk et al. synthesized and characterized Meloxicam-loaded PLGA NPs and later studied their efficacy on HT-29 cells. NPs increased the stability of meloxicam and also helped in a sustained release. The formulations showed a higher uptake and had a higher cytotoxic effect on the cells [122]. Schleich and group incorporated dual agents (Paclitaxel and superparamagnetic iron oxide) in PLGA NPs. High cytotoxicity was observed when PTX-encapsulated PLGA NPs were used, but no such toxicity was noticed in iron oxide-loaded NPs. Both NPs showed a high uptake in CT 26 cell lines. In vivo studies showed delayed regrowth of CT26 tumors [123].

Cooper et al. synthesized and optimized Diclofenac-loaded PLGA NPs by employing varying stabilizers and other parameters. The formulated particles showed a low size (<200 nm), high drug entrapment (80%), and high stability [124].

Rafiei et al. developed docetaxel-encapsulated PLGA NPs for intravenous applications. Surface-modified PLGA NPs (PLGA-PEG) were synthesized, and animal model studies showed that both NPs increased the circulation time and concentration of the drug in the blood [125].

Afrooz et al. co-encapsulated PTX and verapamil (VIR) in PLGA NPs and characterized the formulations for drug loading, zeta, and size. The co-encapsulated NPs showed higher cytotoxicity on MCF-7 cell lines after three days than the free drug [126]. Similarly, Ahmadi et al. co-encapsulated Doxorubicin (DOX) and VIR, which resulted in a similar effect on the MCF-7 cell line [127]. Vakilinezhad et al. co-encapsulated methotrexate and curcumin in PLGA NPs and optimized the formulations. The NPs showed higher cytotoxicity on SK-Br-3 cell lines when compared to the free drug, and in vivo studies also indicated the inhibition of breast cancer [128].

Surface functionalization in polymer-based NPs is the process of altering the existing polymer surface’s physicochemical properties by introducing different materials (hydrophilic polymers) or molecules to enhance its efficacy or to help provide functional groups for further ligand attachments. Surface functionalization methods mainly depend on the surface chemistry and type of ligand, and the NP preparation technique. These are broadly classified under three essential categories, with the interaction between the NP surface and ligand forming the categorization basis. 

In the chemical conjugation method (coupling), the ligand and polymer undergo chemical modifications to attach active groups [129,130] with the help of coupling reagents such as EDC, DCC, and NHS.

The noncovalent methods employ the affinity between the ligand and the NP surface to achieve the desired results. Proteins such as streptavidin, having a high affinity to biotin, are incorporated in the NP to noncovalently attach the ligand to the NP [131,132]. Electrostatic interaction or the physical adsorption method are achieved by selecting the appropriate ligand and NP polymer to promote hydrophobic interactions or hydrogen bonding. The NP can also be coated with surface polymers to mediate the interaction between the ligand and the NP [133,134,135].

Various polymers, such as PEG, PVA, dextran, and chitosan, have been utilized for surface functionalization. PEG is a hydrophilic polymer that has extensively been used due to its ability to protect NPs (via stealth) from rapid renal clearance. Simultaneously, PEG’s presence hinders the interaction that is necessary for uptake in the cells [136]. For this reason, noncovalently bonded PEG (cleavable) on the surface of polymer nanocarriers is preferred, because it not only increases circulation time, but also does not hinder drug release and uptake.

The process of attaching PEG, covalently or otherwise, onto polymer molecules, drugs, and macrostructures is known as PEGylation. PEGylation is a standard method employed to increase the stability of different polymer carriers, drugs, and proteins. The addition of PEG has been shown to improve the efficacy of the carriers and drugs.

Ruan et al. employed triblock copolymers of PLA and PEG to load the hydrophobic drug PTX in microspheres of PLA-PEG-PLA. Their work showed that the release of the drug was faster in PEGylated NPs than regular PLGA NPs. Overall, about 50% of drug release was possible in a sustained manner for a period of one month [137]. Similarly, Danafar et al. encapsulated both hydrophobic and hydrophilic drugs into NPs of PLA-PEG-PLA and obtained high encapsulation efficiencies. The drug release profile was biphasic for the hydrophobic drug and triphasic for the hydrophilic drug [138]. Similarly, Dong et al. formulated, characterized, and optimized PTX-loaded MPEG-PLA NPs that took on the core-shell structure and released the drug in a biphasic manner [139].

Danhier et al. studied the effect of PTX encapsulated in PEGlylated PLGA NPs on HeLa cell lines compared to other commercial formulations, such as Taxol and Cremophor EL. The results showed that treatment with PTX-loaded NPs leads to lower viability when compared to the other formulations. In vivo studies also indicated more significant growth inhibition of tumors with the PTX-loaded NPs [140].

Rafiei et al. synthesized, characterized, and optimized Docetaxel-loaded NPs of PLGA and PEGylated PLGA, and found that the drug’s release was higher in PEGylated NPs. The higher encapsulation also enhanced the blood concentration of the drug during in vivo studies [141].

Sims et al. used PEGylated PLGA NPs, in addition to other surface-functionalized PLGA NPs, and studied the internalization efficiencies on HeLa cell lines. They found that coating with PEG reduced cellular internalization but increased tissue penetration [142]. In vivo studies of PEGylated PLGA NPs encapsulating curcumin synthesized by Khalil et al. showed that curcumin release was slower in non-PEGylated NPs. Nonetheless, the bioavailability of the drug was significantly higher when delivered through PEGylated NPs. Both NPs were able to increase the mean half-life of the drug [143].

Interfacial activity assists in the surface functionalization of polymer-based NPs, and molecules such as PEG increase the circulation time of the NPs. However, this process is accompanied by a number of drawbacks [144]. Receptor-mediated targeted drug delivery helps overcome most of these issues and is also beneficial, because normal cells/tissues are left unaffected. Attaching ligands onto the NPs requires functional groups that are lacking in polymers such as PLGA. However, PEG can be modified to possess homo- or hetero-bifunctional groups [145]. This process provides the opportunity to attach the desired/possible ligands onto the PEGylated NPs, drugs, or other macromolecules.

Attaching ligands to polymer-based carriers is often performed through chemical conjugation, which requires in-depth knowledge and proper control of all of the variables (ligand, polymer, drug, process variables), which is an expensive and time-consuming [145,146,147] process. The interfacial activity-assisted surface functionalization technique utilizes the interfacial activity of amphiphilic polymers to incorporate the ligand into the polymer carrier, such as PLGA. The process of functionalization is based on the principle of self-assembly. The hydrophobic part (PLA) of a block copolymer (PLA-PEG) is inserted into the PLGA NP. At the same time, the hydrophilic PEG chain with the ligand remains on the outer surface of the PLGA NP during the solvent evaporation step of NP synthesis. Patil et al. simultaneously functionalized PTX-loaded PLGA NPs with folic acid (FA) and biotin through the PEG-PLA block copolymers. The drug-loaded NPs showed better efficacy on numerous cancer cell lines, and studies also indicated an enhanced accumulation of these NPs during in vivo studies [147].

Similarly, Toti et al. utilized the maleimide end group in their block copolymer of PLA-PEG to conjugate the cRGD peptide, which was then attached to the coumarin 6-loaded PLGA NPs. A significant and high cellular uptake was observed on numerous cell lines. Their study showed a two-fold increase in conjugate-NP accumulation during in vivo studies compared to NPs without the ligand [148].

Roger et al. also employed the same method to conjugate the FA moiety to PTX- loaded PLGA NPs. Cell line studies on Caco-2 showed a five-fold increase in the apparent permeability of PTX when encapsulated in the PLGA polymer carrier. The FA-functionalized NPs underwent an eight-fold rise in transport compared to the free drug, thereby increasing the drug’s oral bioavailability [149].

The IAASF technique was also used by Dhoke et al. to conjugate Lactosaminated- Human Serum Albumin peptide on PLGA NPs, loaded with the drug lamivudine. In vivo studies also exhibited the benefits of ligand-based targeting [150].

The targeting of diseased sites/tissues through the ligand–receptor route has gained significant attention in recent decades. Drug delivery by this method not only protects healthy cells, but also helps in increasing the therapeutic effect. The effective use of polymers to carry the drug adds to the efficacy, because sustained drug delivery is possible. Studies have shown that cancer cells overexpress specific receptors and thus can be utilized to deliver the drug. Similarly, activated macrophages also overexpress numerous receptors, including folate (FRβ) [151].

Folate receptors (FRα and FRβ) are among the many receptors known to be over- expressed on cancer cells. Folic acid, a synthetic version of vitamin B9, is known to show affinity towards these receptors. Many researchers have utilized this ligand–receptor route to target and deliver the associated drug [152]. Vortherms et al. conjugated folic acid to PEG, which was later coupled to 3′-azido-3′-deoxythymidine (AZT). In vitro cytotoxicity studies on the A2780/AD cell line that overexpressed folate receptors showed a 20-fold increase in potency compared to free AZT [153]. Xiong et al. synthesized FA-PEG-PLA block copolymers for targeted delivery of PTX on KB cell lines. Cytotoxicity studies indicated that the folic acid conjugated micelle toxicity on the cells continued to increase with the folate content. Cells treated with the free drug displayed lower toxicity than PTX-loaded FA-PEG-PLA micelles [154].

Similarly, Hami et al. synthesized and characterized folate-functionalized PLA- PEG block copolymer micelles and later conjugated DOX to these micelles. Cytotoxicity studies on SKOV3 human ovarian cancer cell lines showed significantly higher toxicity compared to non-targeting micelles [129]. Goren et al., in early research, demonstrated that folic acid conjugated liposomes loaded with DOX showed 10-fold higher toxicity on M109R cells compared to unconjugated liposomes. Inhibitory effects in vivo also indicated a significantly higher effect compared to free DOX [155].

Chandrasekar et al. synthesized folate-dendrimer conjugates to deliver indomethacin to inflammatory regions. The study showed that drug encapsulation increased with an increase in folate content. The drug’s half-life increased, and drug exposure to the site was also significantly higher for folic acid-conjugated PAMAM dendrimers compared to the unconjugated formulation [156]. Zhang et al. and Pan et al. conjugated FA to d-α-Tocopherol polyethylene glycol succinate (TPGS). They compared it with other polymer carriers loaded with drugs, such as PTX and DOX. The cytotoxic effects of FA-conjugated carriers on C6 cell lines showed significantly higher inhibition and increased uptake in MCF-7 cells, thereby highlighting the benefits of receptor-mediated targeted drug delivery [157,158].

GCs are steroid hormones released in the body when stimulated by stress. They primarily function as an anti-inflammatory agent and also as an immunosuppressant. They cause severe side effects when deployed in large doses. Some of the most prominent synthetic GCs are DEX, cortisol, and prednisolone. GCs are predominantly used to treat inflammatory conditions, such as asthma and rheumatoid arthritis. They have also been used as a supplementary drug to reduce edema in cancer tumors. Recent studies have indicated the cytotoxic effects on glioma and other cancers [159].

Morita et al. investigated the effect of DEX on C6 cells and found that serum deprivation led to cell death, but DEX’s presence further enhanced necrotic death in the glioma cells [160].

Shapiro et al. and Grasso et al. studied the effect of various GCs, such as cortisol and DEX. They concluded that GCs primarily inhibited glioma cell growth [161,162,163] but sometimes displayed transient inhibition [164]. Gurcay et al. presented the initial studies on prednisolone’s effect on primary brain tumor and identified growth inhibitory effects [164].

Kaup et al. studied the inhibitory effects of DEX on different glioma cell lines, such as A172, T98G, and 86HG39, to elucidate the effects of GCs on tumors. The cell lines were subjected to acute (continuous), pre, and combination (pre and acute) treatments. A time-delayed inhibitory effect was noted in all cell lines when subjected to DEX pre- treatment. A172 and T98G cell lines displayed significant inhibitory effects when subjected to combination treatment. Acute treatment of DEX had potent inhibitory effects on A172, whereas the effect was negligible on T98G and 86HG39 cell lines [165].

Fan et al. reported a concentration-dependent inhibitory effect of DEX on murine and rodent glioma tumor growth. It was also reported that primary astrocytes (human) and primary neurons (rodent) were not affected by DEX [166].

### 1.3. Physicomechanical, Thermostability, and Morphological Characteristics of Biodegradable Polymeric Materials

Polyolefins such as polypropylene (PP) and PE are resistant to biodegradation. It has been observed by numerous authors [167,168,169] that these polymers are not significantly affected by soil burial, whereas Darby [170] and Griffin [171] found that PE is successively degraded in compost. Moreover, they established a relationship between the decrease in the tensile strength and the extent of biodegradability. However, molecular weight was the critical factor in this assessment. Studies with polyethylene samples indicated that bacterial growth decreased with the increase in molecular weight [172]. Potts et al. [173,174,175,176] conducted studies on the biodegradation of synthetic polymers, such as polyester, PE, and PS. They found that, among these high molecular weight synthetic polymers, only the aliphatic polyesters and oligomers of PE showed biodegradability, whereas PS did not. Weiland [177] and Khabhaz et al. [178] reported degradation of thermally oxidized PE. It is well known that the low molecular weight polymers and straight chain polymers are more affected by microbial degradation [177,179,180]. Further branching and crystallinity [181,182] have also been observed to reduce the rate of biodegradation.

For biodegradation to take place, some simple organic substances should be added to polymers for rapid decomposition. Photolabile chemical groups (e.g., carbonyl moieties and benzophenone) have been introduced into polymers to accelerate their UV light catalyzed depolymerization in the environment via the free radical process [183,184]. Products containing photosensitizers are affected by light and become biodegradable thereafter. For certain applications and disposal routes, this may be a viable option. However, in many cases the materials will not be exposed to sunlight when discarded or buried, and coatings may obscure the direct exposure to the light that is necessary to initiate the degradation process. Hence the aforesaid approach remains questionable.

In addition to the already-cited polymers, acrylonitrile-butadiene-styrene tercopolymer (ABS), aromatic polyesters (including PET, polyether-urethanes, and most acrylates except poly(alkyl alpha-cyanoacrylate)) are also considered to be resistant to biodegradation.

Although polyethylene (PE) is considered to be resistant to biodegradation, Lee et al. [185] reported biodegradation of PE by phanerochaete and streptomyces species in blends containing 6% starch and oxidants. They reported a decreased molecular weight and changes in mechanical properties due to biodegradation. However, only low molecular weight fragments appeared to be responsible for the observed biodegradation. A degradable PE–starch complex has also been prepared using a transition metal and an antioxidant [186,187].

Blown films containing up to 40% starch have been developed by extrusion and the biodegradability was assessed by measuring the changes in weight loss and chemical composition by FTIR [188,189,190,191,192,193,194,195,196]. However, Psomiadou et al. [197] reported that more than 30% starch had deleterious implications for the mechanical characteristics of LDPE/starch films used for food packaging [198]. 

A number of degradable blends of starch and PE have been previously synthesized [199,200,201], and their environmental weathering [202] and biodegradation has been studied in a compost environment [203], soil [204], and by microbial culture [205,206]. Thermoplastic PE/starch compositions were reported to have the required strength and biodegradability [207,208], and those developed by Chiquet [209] were found to decompose during heating, exposure to UV light or sunlight, and composting. 

Chandra and Rustgi [210] investigated the biodegradation of maleated LLDPE and starch blends. They observed that the tensile strength and moduli escalated and percent elongation-at-break reduced as the starch concentration in the mixtures increased. Poly(ethylene-co-acrylic acid) has been frequently used as a compatibilizer in starch composites with LDPE [211,212], HDPE [213], and blown films [214,215,216] of PE, which have been applied in agricultural mulch and in packaging. Li et al. [217] conducted studies on a starch graft copolymer as a compatibilizer for LLDPE/starch. Many commercial products containing LDPE and starch have appeared in recent years [169,218,219,220].

**Figure 6:**
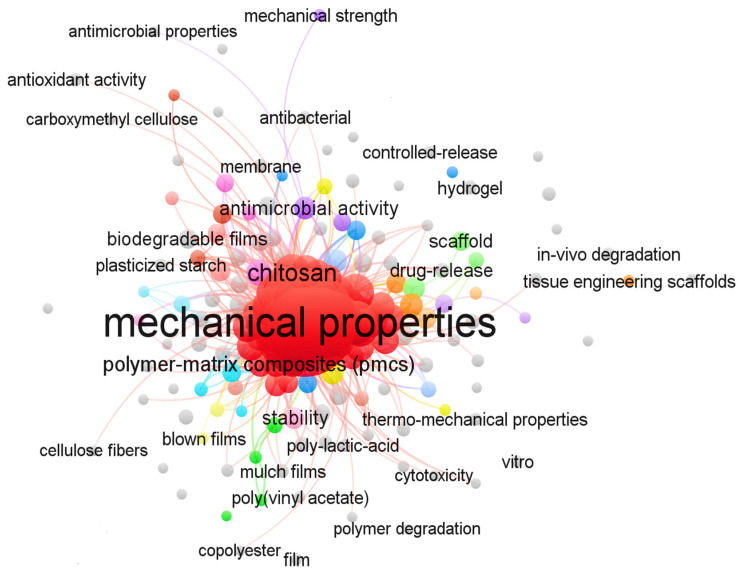
Systematic mapping summary of scientific advances in physicomechanics and thermostability in in vitro drug release studies of biopolymeric materials/biocomposites for biomedical applications.

A number of reports are available in the literature in which starch modified by various methods was used for the development of biodegradable products. Japanese patents have been filed [221,222,223,224,225] on the production of biodegradable polymeric thin films with superior physicomechanical characteristics utilizing starch derivative compounds. Favis et al. [226] obtained a patent for a composition containing LDPE, alkyl ethers of starch, and a vinyl or acryl polymer as a compatibilizing agent that showed good biodegradability, as exhibited in Figure 6.

Shah et al. [227] investigated the initial degrading process of starch-filled LDPE strips compounded with commercially available, well-dried, modified, granular starch (CATO-32). Numerous ecological situations have been reported to have combinatorial synergism impacts on the decomposition rate and processes. Various new technologies have been developed for the manufacture of biodegradable starch PE blends. Muller et al. [228] carried out oil-philicity treatment of starch with a coupling agent, which promoted uniform dispersion of starch in PE and improved the mechanical properties of the blends. Pierre et al. [229] devised a method for combining the blends containing thermoplastic starch (gelatinized starch plasticized with glycerol) in a continuous manner in a co-rotating dual-screw-based extrusion process.

The biodegradation of PE films containing 40% gelatinized corn starch and 15% EAA were investigated in a diverse spectrum of aquatic conditions [230]. The loss-of-starch on degradation was preceded or followed by reduction in the tensile strength, and enabling of the films to disintegrate to test their susceptibility to mechanical strain. By comparison, Shogren et al. [231] reported that films with the same composition exhibited a heterogeneous microstructure with a non-uniform distribution of starch. The compatibility, physicomechanical characteristics, and morphology of LDPE-starch/modified-starch blends used in agricultural mulch and packaging films were studied by Tian et al. [232] for the films. Similar starch-PE agricultural mulch was prepared using a graft copolymer of starch and MMA [233]. The manufactured films showed better biodegradability but a higher production cost than conventional PE [234]. 

A detailed study on the characteristics of various fatty acid esters of starches and their mixtures with LDPE was carried out by Bikiaris et al. [235] and Thiebaud et al. [236]. The latter observed that the thermal stability and elongation increased, but the tensile strength and water absorption decreased on esterification. Aburto et al. [237] examined the characteristics of octanoated starch and its mixtures with PE. Its blends with LDPE showed better mechanical properties and thermostability, and lower water absorption in comparison with the mixtures of LDPE with plasticized starches. Starch modified by various fatty adds, such as lauric acid, palmetic acid, and stearic acid, has been used to develop bio- and photodegradable PE films [238].

Evangelista et al. [187] investigated the impact of compound blending and starch alteration on the characteristics of starch-filled LDPE. They observed that the cast films of LLDPE comprising starch-octenyl-succinate revealed high tensile strength and percent elongation, but a lower rate of biodegradation than those containing native corn starch. Kshirsagar et al. [239] synthesized starch acetate with varying acetyl content. They investigated the rheology and permeability characteristics of blends of starch and starch acetate with LDPE.

The other synthetic polymers used for blending with starch are polypropylene (PP), polyvinyl alcohol (PVA), polyvinyl acetate (PVAc), some modified polymers, and copolymers [240,241,242,243,244]. 

The effect of operating factors on physical characteristics of blends of starches with ethylene-vinyl-acetate (EVA) and polyethylene modified with maleic-anhydride (EMA) was observed by Ramkumar et al. [245]. Rheological and morphological analysis of corn-starch and SMA/EPMA mixtures containing 60–70% by weight of the starches was undertaken by Sethamraju et al. [246].

Otey et al. [247] formulated starch-based PE films comprising up to 40% starch using urea and ammonia. Fanta et al. [248] also discussed the effect of urea in the presence of water or aqueous ammonia on the composites. Reis et al. [249] reported the in vitro decomposition of starches/ethylene-vinyl-alcohol polymeric-blends and observed a distinct rheological behavior and mechanical properties. The same authors also carried out a detailed investigation on starch–EM composite films.

Polymer compositions that yield films with good strength [250] and migration-resistant plasticizer [251] are obtained from starch/poly(ethylene-co-vinyl alcohol) [252,253,254]. Starch-PVA blends [255,256,257,258,259] are useful as moisture-resistant biodegradable polymers for agricultural mulch, shock absorbing foams, and articles with good dimensional stability.

Starch-based biodegradable packing materials have been developed by extrusion of starch with polyethylene glycol [260] or PVA [261]. Such films have been prepared by mixing starch with poly(vinyl alcohol) [262], vinyl acetate and its copolymers [263,264,265,266,267,268,269,270,271,272,273,274,275,276], and EM. The films also exhibited excellent mechanical strength and flexibility.

Polymer compositions containing starch have also been formulated using bacterially produced poly-3-hydroxybutyrate (PHB) and poly-3-hydroxyvalarate (PHV). Roller and Owen carried out studies on the structural and physicomechanical characteristics of melt-pressed sheets of PHB and PHV filled with various amounts of particulated maize starch granules. 

Processing and mechanical properties, and biodegradation, in municipal activated sludge of starch-poly(p-hydroxyl butyrate–covalerate) composites have been extensively studied. The change in composition after biodegradation was quantified by FTIR, and increased starch content was observed to result in more extensive degradation. Lactic acid copolymer-starch compositions useful for films, filaments, and packing materials were found to be biodegradable. It was observed that the film was broken into pieces after two weeks and disappeared after two months.

PP-based biodegradable plastics and blends have been prepared by mixing starch with maleated PP. Some ternary blends of starch and PP with polylactide and ethylene-vinyl acetate copolymer have been reported for disposable diapers, and with PE for the marine environment [276].

Bennett et al. [277] developed a rigid urethane foam formulation containing 10 to 40% starches. These investigations revealed that foams prepared from starch-derived products show better flame resistance but are readily attacked by soil microorganisms.

The incorporation of surface-altered starches to plastics has been shown to be a commercially feasible means of producing conventional biodegradable polymeric materials [278]. Starch in various forms, such as starch xanthate, gelatinized starch, and dried starch, has been incorporated in large amounts as a filler in disposable PVC-based plastics [279,280].

Several modified forms of starch, such as silyl isocyanate modified starch dialdehyde [281], acid hydrolyzed starch [282], and starch derivatives [283,284], have been used to develop thermoplastic mixtures by blending with other plastics. Jeremic et al. [285] developed blends of thermoplastic starch and thermoplastic polymers, such as EM and EVAc, in a twin-screw extruder [286].

Biodegradable plastics with high tensile strength have been obtained from PE, PP, PS, and PVC with 6 to 50% of octenyl succinate starch metal ion complexes [287]. Similarly, biodegradable starches containing polymer compositions with good mechanical properties were developed by Osada et al. [288].

Huang et al. [289] carried out an in-depth study of the development of the technology and product application of the biodegradable plastics based on graft copolymers of starch. Imam et al. [290] studied the morphological and thermo-behavior of poly3-hydroxy-butyrate hydroxy-valerate/starch valerate mixtures, and reported that no phase separation was observed.

Several patents based on the use of modified starch for blending with synthetic polymers are available. Biodegradable foams useful as packing material were patented by Jaffs [291]. Bastioli et al. [292,293,294] developed a composition for biodegradable plastic moldings based on destructured-starch and blends of starch with plastics for strong films, sheets, and fibers. The preparation and use of a biodegradable starch derivative and polymer mixture are discussed in a European patent [295]. Berruezo [296] developed photo/biodegradable high impact polystyrene (HIPS) sheets by mixing modified starch, HIPS, and photo-degrading agents.

### 1.4. Comparative Analysis of Physicomechanical, Thermostability, Rheological, and Morphological Characteristics of Biopolymeric Materials with Other Materials

**Figure 7:**
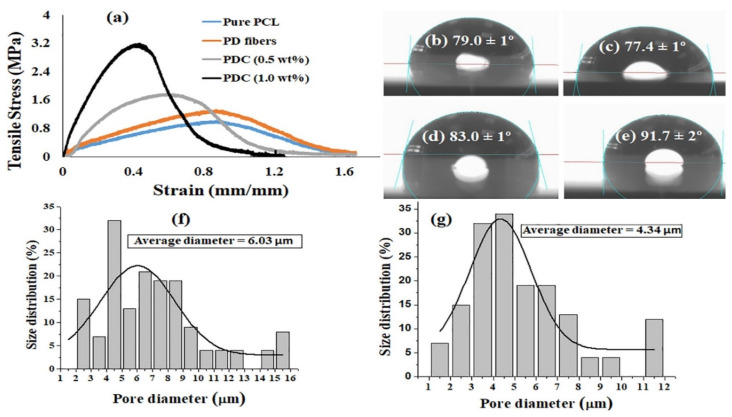
(**a**) Tensile graphs; (**b**–**e**) contact angles of neat PCL, PD, PDC (0.5 wt.%), and PDC (1.0 wt.%); and (**f**,**g**) porosity/permeability of PDC (0.5 wt.%) and PDC (1.0 wt.%) composite filaments. Reproduced with permission from [297].

In another study, Mamidi et al. [297] presented a new class of PCL/f-CNOs composite. Newly developed composites were characterized using SEM and FTIR techniques. The DOX releasing ability was analyzed under various pH conditions. The authors reported that force spinning provides regular and bead-free nanofibers in the range of 210–596 nm. Results indicated a good control over the DOX release in forcefully spun PCL/f-CNOs fibers, in addition to better tensile strength (3.16 MPa). These results reflect the stability, viability, and cell adhesion characteristics of the reinforcement in the matrix material, as depicted in Figure 7. The newly fabricated composite is used in numerous applications in the biomedical field. 

**Figure 8:**
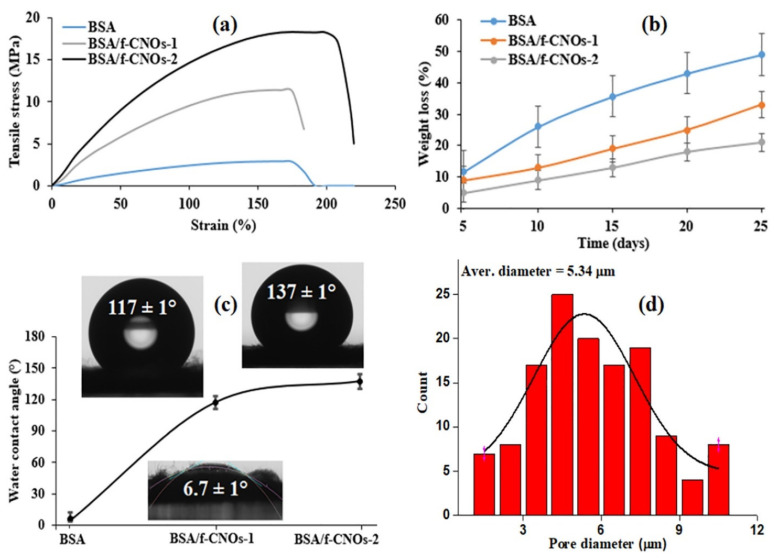
(**a**) Tensile strength; (**b**) degradation; (**c**) water contact angle for virgin BSA, BSA/f-CNOs-1, and BSA/f-CNOs-2 filaments; (**d**) porosity/permeability of biomimetic or biocompatible BSA/f-CNOs-2 nanocomposite fibrils. Reproduced with permission from [298].

Mamidi et al. [298] prepared PHPMA-CNOs = f-CNOs reinforced BSA nanocomposite fibers using the force-spinning technique with DOX as a drug. The DOX-releasing ability was measured in terms of the concentration of DOX, the incubation temperature, and the pH value. The authors observed 72–95% drug release at the temperature limits of 37–43 °C in fifteen days of study. The mechanical strength of the BSA increased to 18.2 MPa with the addition of f-CNOs, in addition to the water absorption angle and thermal characteristics revealed in Figure 8. 

Mamidi et al. [299] prepared PHPMA-based composites that were reinforced with SWCNT. The composites were primed using force spinning followed by the thermal pressure approach. The newly developed composites possessed a tensile strength of 13.7 GPa, an elastic modulus of 243.3 GPa, and a toughness of 1421 J/g. The authors reported that the newly developed composites were the strongest and stiffest composites among all of the nanocomposites available in the literature. 

Mamidi et al. [300] adopted the force-spinning approach to develop 3D scaffolds of gelatin and zein protein. The authors reported that one unit of gelatin and four units of zein provided better tensile strength and hydrophobic behavior at a water angle of 115°. 

**Figure 9:**
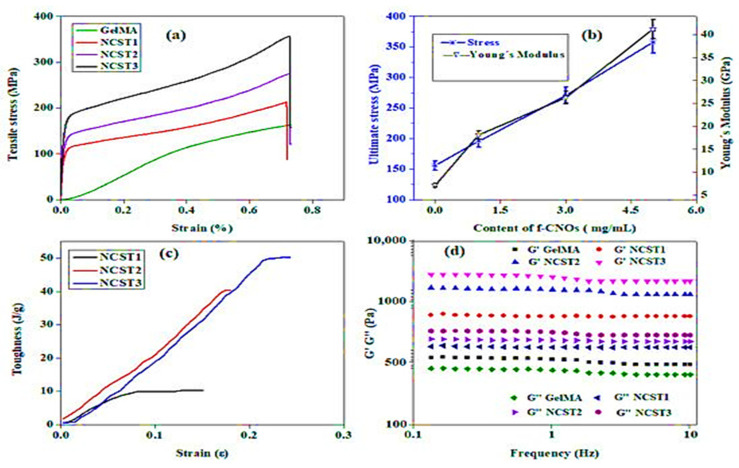
Graphs demonstrating hydrogel physicomechanical characteristics: (**a**) tensile stress; (**b**) tensile strain versus elastic moduli; (**c**) impact strength; and (**d**) storage and loss modulus (G′ G″). Reproduced with permission from [301].

Mamidi et al. [301] developed PAPMA-CNOs and (GelMA)/f-CNOs/CD supramolecular hydrogel interfaces using the photo cross-linking approach. The morphology and properties were evaluated. The authors observed that the maximum drug release occurred under acidic conditions over 18 days. In addition, (GelMA)/f-CNOs/CD composites exhibited better properties than the alternative composite. The physical and chemical behaviors, microstructure, biodegradation, and swelling characteristics of hydrogels have been studied. Over an 18 day period, the composite hydrogels exhibited improved controlled release of the drug under acidic environments (pH 4.5 = 99% and pH 6 = 82%). GelMA/f-CNOs/CD supramolecular hydrogels possessed significantly enhanced tensile strength (ultimate strength = 356.1 ± 3.4 MPa), impact strength (K = 51.5 ± 0.24 Jg^−1^), and elastic moduli (E = 41.8 ± 1.4 GPa) with the addition of f-CNOs. The framework of GelMA/f-CNOs/CD hydrogel augmentation reveals an excellent distribution and extent of polymeric envelopment of f-CNOs throughout GelMA matrices, as displayed in Figure 9. Additionally, the produced hydrogels were found to have significant cell viability when evaluated against human fibroblast cell cultures. Nonetheless, the primed supramolecular hydrogels will form the basis of future targeted drug carrier systems utilizing regulated delivery methods. 

Cole et al. [302] evaluated the mechanical properties of the exopolysaccharide biopolymers by developing methods to obtain biopolymer bonding among the grains of natural occurring materials. The authors examined two treatments in their experiments. The first treatment refers to one week of incubation at 28 °C in yeast extract mannitol media. The purpose of this treatment was to determine cohesive and adhesive properties of the polymer. The second treatment related to the precipitation and resolubilization of the Rhizobium EPS from the media supernatant. The authors observed an increased bond stiffness with an increase in curing time. The modulus was varied within the limits of 0.2 to 3.2 MPa, and cohesive strength was varied from 16 to 62 MPa. In addition, the cohesive strength of the precipitated exopolysaccharide biopolymer was more than that of natural exopolysaccharide.

**Figure 10:**
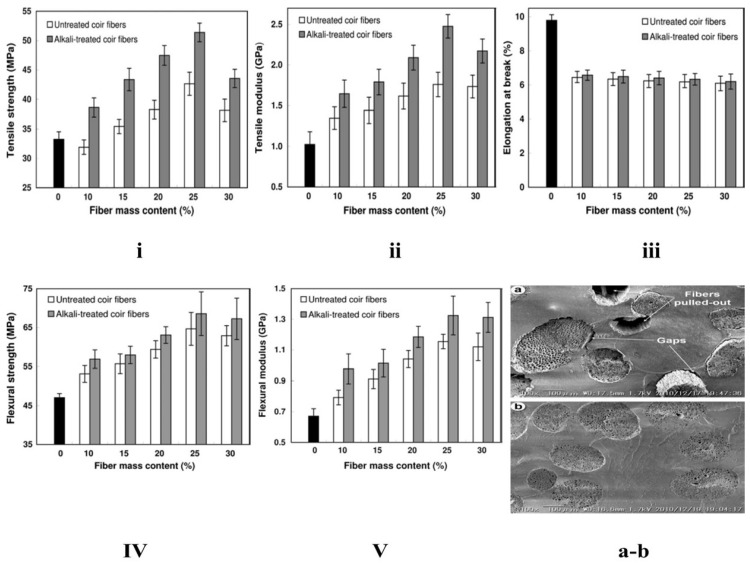
(**i**) Tensile strength; (**ii**) tensile moduli; (**iii**) fracture strain; (**IV**) moduli of rupture/bending strength; (**V**) bending modulus of unprocessed and 5N-72-treated coir-fiber/PBS biodegradable composites; and tensile fracturing surfaces of PBS biodegradable composite strengthened with 20% weight concentration of: (**a**) unprocessed coir fibers, (**b**) 5N-72 processed coir-fibers. Reproduced with permission from [303].

Nam et al. [303] developed PBS biodegradable composites that were embedded with coir fibers. The influence of alkali treatment on the properties and morphology was analyzed. The highest shear strength was observed in a sample that was soaked in 5% NAOH solution for 72 h at room temperature. In addition, the mechanical properties were improved significantly with the alkali treatment. The composite containing 25% coir fibers resulted in increases of around 55, 142, 46, and 97% in tensile strength, tensile modulus, flexural strength, and flexure modulus, respectively, as shown in Figure 10. The morphology results indicated better interfacial bonding in alkali-treated composites than untreated composites. 

Treated natural fiber materials have the capability to replace synthetic fiber materials because they are more easily available, less expensive, ecofriendly, renewable, and light weight. Among the available natural fibers, kenaf possesses excellent properties, and jute fiber exhibits high strength and compatibility with biopolymers [303].

**Figure 11:**
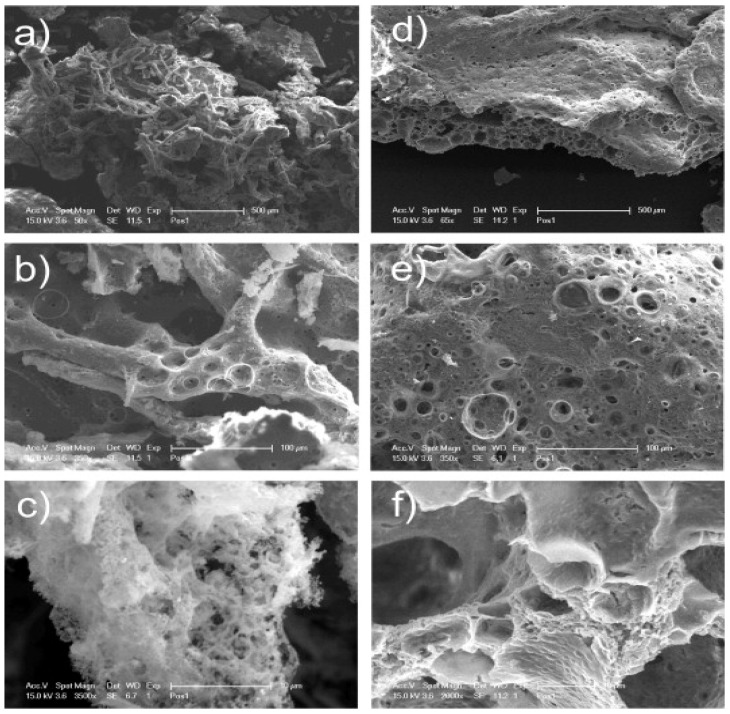
Microstructure of layered 2 (**a**–**c**) and Lam 1:6 (**d**–**f**) under numerous varied magnification scales. Reproduced with permission from [304].

Gallo et al. [304] fabricated kenaf fiber-reinforced biopolymer composites using compression molding. The influence of core thickness of the fibers on the properties of polyhydroxyalkanoates was evaluated. Kenaf-reinforced material prevents the combustion of the material and kenaf performs as a carbonizing compound, which provides an insulating layer on the material’s surface. Results revealed that monofiber-reinforced composites possess better mechanical properties than double-layered composites. The regular dispersion of the kenaf material in the matrix ensures improved properties of the composite, as shown in Figure 11. The dense microstructure obtained from fire residues indicates that the reinforcing material acts as a carbonizing agent. 

**Figure 12:**
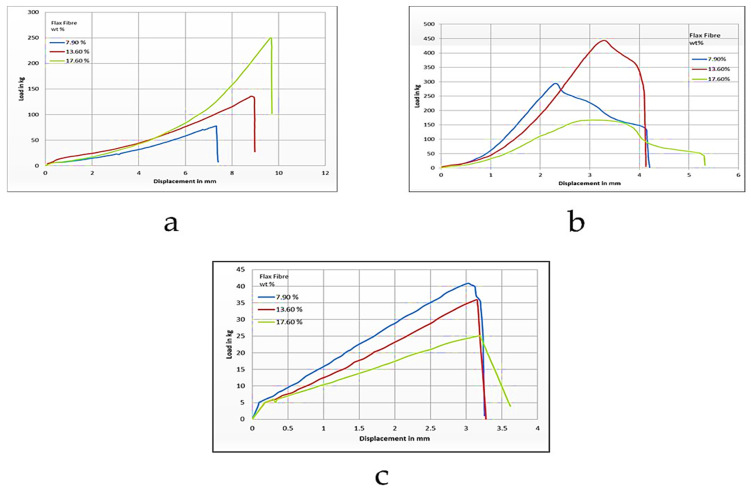
Loading versus displacement graphs from: (**a**) tensile test; (**b**) compression test; and (**c**) bending test. Reproduced with permission from [305].

Motru et al. [305] developed flax-reinforced PLA polymer matrix composites. During fabrication, flax fibers were varied by weight, in the values of 7.9, 13.6, and 17.6%. The authors observed that mechanical properties were increased with an increase in the fiber content. In contrast, for treated PMCs, the opposite results were observed. The flexural strength remained the same for all of the composites, whereas the maximum UTS was observed in a composite containing 13.6% flax fibers. The impact energy of the newly developed composites was varied within the limits of 25–30 joules, as exhibited in Figure 12. The authors also observed a regular dispersion of the flax fibers within the PLA matrix in all composites.

Russo et al. [306] fabricated kenaf-reinforced polymer matrix composites with PHBV, LDPE, and PBAT as matrices using melt compounding. Kenaf fibers were treated chemically before being added to the matrix to enhance interfacial bonding among the constituents. Cast composites were alkalized and silanized to improve their performance. Thermal properties of the PHBV were not altered due to the addition of kenaf; however, a negative effect was observed in the case of PBAT. In addition, PHBV-based composites possessed better flexural, modulus, and impact strength, irrespective of the type of treatment performed. However, the existence of stiff cellulose fibers reduced these properties in PBAT- and LDPE-based composites.

Alvarez et al. [307] prepared sisal fiber-reinforced polymer matrix composites. The composition of the reinforced fibers was varied from 5 to 15% by weight. The water sorption ability of the sisal, starch, and their composites was evaluated by varying the reinforcement compositions. The authors reported that sisal fiber absorbed less moisture than the base matrix; however, the diffusion coefficient value was approximately same for all fibers. In addition, the fibers possessed a higher diffusion coefficient than the base matrix. The diffusion coefficient was marginally increased with increasing fiber content, and the moisture content had a harmful effect on the mechanical behavior of the composites. The flexural modulus was reduced with an increase in fiber content. 

Liang et al. [308] developed kenaf-reinforced PBS polymer matrix composites using a mixing process. The mechanical behavior, morphology, and crystallization behavior were evaluated. The composition of kenaf was varied from zero to 30% by weight in steps of 10%. The authors reported that the moduli and crystallization rate were increased with the addition of kenaf material to the base matrix. The authors also observed that the tensile strength and storage modulus of the newly developed composite were increased by 53 and 154%, respectively, with the addition of 30% kenaf to the base matrix and the increase in the crystallization temperature from 76.3 to 87.7 °C. SEM result illustrates that further improvement at the PBS and kenaf interface is required to increase the interfacial bonding. 

Zhu et al. [309] blended sisal fiber-reinforced PLA composites. Initially, hybrid sisal fibers were prepared by mixing treated and untreated sisal fibers and then introduced into the PLA matrix. The authors reported an enhancement in the crystallinity and the impact strength of the base matrix with the addition of sisal fibers due to β crystals, as observed in XRD patterns. It was also reported that properties of the hybrid sisal reinforced composites were significantly improved, with the treated and untreated sisal-reinforced composite and PLA/HSFs composites possessing 47.1% and 30.8% higher strength and crystallinity, respectively, than the other samples.

Jung et al. [310] investigated the effect of chemical bonding on the mechanical behavior of biopolymer composites. The behavior of these bonds under mechanical deformation was also evaluated. The authors observed that hydrogen bonds enabled high deformation and changed the structure under low loading conditions. In addition, mutation caused a change in the stable structure of the biopolymers. 

Bahrami et al. [311] reviewed the mechanical behavior of hybrid biocomposites. The properties reviewed by the authors included the strength, water absorption, and flammability of the biocomposites. It was found that the use of hybrid fibers and the treatment of the fibers, among other factors, have a significant effect on the mechanical behavior of the biocomposites. The authors reported that biocomposites will be superior to numerous alternative engineering materials in the future. 

Kremensas et al. [312] developed wood fiber-reinforced biopolymer matrix composites boards. The authors used corn starch and hemp shiv treatment during the production of biocomposites. The results revealed that compressive, tensile, and bending stresses of 3, 0.45, and 6.3 MPa, respectively, were obtained at 10% hemp shiv by weight. In addition, the composite containing 10% corn starch by weight exhibited better a contact zone and increased the product strength. 

**Figure 13:**
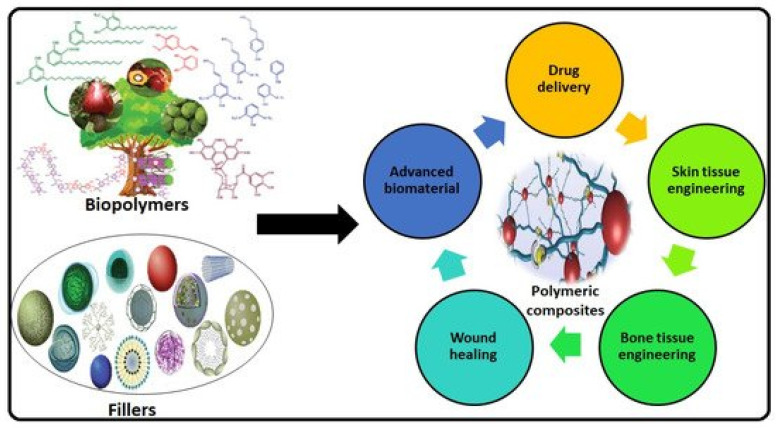
A graphical description highlighting the numerous promising functionalities of biodegradable polymerics with numerous filler particles. Reproduced with permission from [313].

Aslam Khan et al. [313] presented a review article on the recent progress of biocomposites for tissue engineering and regenerative medicines. Biopolymers are used in numerous applications in wound healing and other medical areas, as reported in Figure 13. Biopolymers are the best alternate among petroleum-based synthetic polymers because they are ecofriendly, environmentally sustainable, and readily available. Biopolymers lack strength and stability, which can be overcome with the use of ceramic-reinforced biopolymer composites. Further study on these materials is required to increase their industrial applications.

Baldino et al. [314] proposed a new process that improves ESPR atomization due to a mixture of SC-CO_2_ in which polymers can dissolve. This occurs due to the reduction of surface tension and viscosity, which enables the production of micro- and nanoparticles of controlled dimensions. 

**Figure 14:**
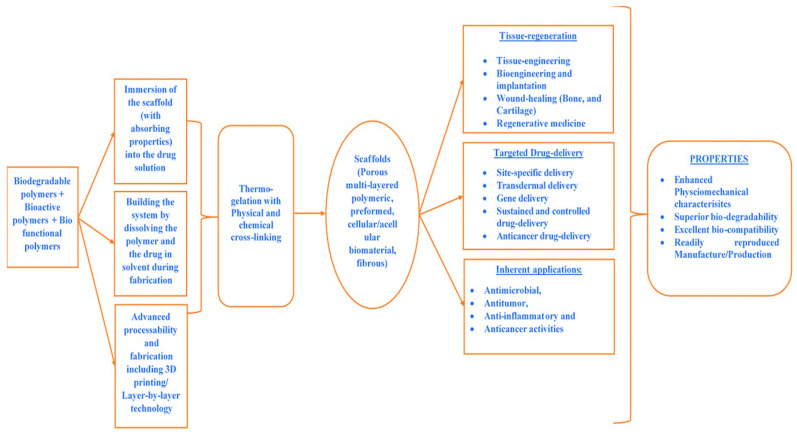
Visualization summarizing the numerous potentially attractive characteristics of biodegradable/bioactive polymerics for biomedical applications.

The above summary provides insights into synthesized biodegradable and bioactive polymeric composites with biological functionality and remarkable compatibility for biomedical application domains, as shown in Figure 14.

Considerable work has been carried out on polymeric-blend materials and copolymers to control crucial aspects of biocompatible polymers, such as degradation rates and physicomechanical characteristics. Moreover, cellular films, implantable biomaterials, three-dimension-printed scaffolds, and nano-structured biomaterials have been produced that acquire the benefits of the biopolymeric stimuli-responsive properties of certain polymerics to improve modulation of biologically active molecule delivery, tissue regeneration, regenerative medicine, and wound healing. Consequently, the current review underlines the critical factors that must be considered in biocompatible hydrogels throughout tissue regeneration and drug carrier delivery; discusses the numerous frequently researched natural and synthetic biomimetic polymeric materials; and provides insight into the developed production and manufacturing methods that employ biodegradable polymeric materials for biomedical applications.

Therefore, this summary examines the contemporary status of biodegradable naturally and synthetically derived polymers for biomedical fields, such as tissue engineering, regenerative medicine, bioengineering, targeted drug delivery, implantation, and wound repair and healing. Furthermore, this review presents insights into a small number of the commonly used tissue engineering applications, including drug delivery carrier systems, demonstrated in the recent findings. Due to the inherent remarkable properties of the biodegradable and bioactive polymers, such as their antimicrobial, antitumor, anti-inflammatory, and anticancer activities, certain materials have gained significant interest in recent years. These systems are being actively researched to improve therapeutic activity and to mitigate adverse consequences. This article also presents the main drug delivery systems reported in the literature and the primary methods for impregnating polymeric scaffolds with drugs, their properties, and the respective benefits for tissue engineering.

## 2. Methodology

The systematic literature review is the most well-known form of literature review and presents a clear picture to the researchers in a more transparent manner. The phases of a systematic review are the identification of articles; screening of the articles according to the established criteria; assessing eligibility according to the content of the articles; and inclusion of the final articles for the analysis. 

### 2.1. Identification

The identification of articles is undertaken in such a manner that it can be reproduced if the search database is given to other researchers. It also allows the review study to be transparent. In this review, we examined the articles related to biodegradable and bioactive polymers, for antimicrobial, antitumor, anti-inflammatory, and anticancer purposes, published during the past 21 years i.e., from 2000 to 2021, using the advanced search option of the Web of Science (WoS) and SCOPUS databases. The searches used for identifying the relevant articles were: “Biodegradable Polymer Nanocomposites for Biomedical applications”, “Natural polymeric biomaterials for tissue-engineering and drug-carrier”, and “Bioactive polymers for biomedical applications”. These specific document searches identified 485 papers that were sent to the screening process.

### 2.2. Screening and Eligibility According to the Relevancy of the Articles

The identification process was followed by the screening process to select the relevant articles according to the theme.

During the process of screening, duplicate articles were removed. Furthermore, some records were also excluded based on article titles that are not relevant to the theme. Finally, the remaining 88 full-text journal papers were further assessed for eligibility based on the content according to the criteria. All of the articles were thoroughly read to determine if they focused on any one of the biomedical applications of the biodegradable polymers or other natural polymeric biomaterials. 

### 2.3. Inclusions

The research articles selected for the review were focused on the performance characteristics of biodegradable and natural polymeric materials for biomedical applications. All of the articles focusing on the performance of biodegradable and bioactive polymers, for antimicrobial, antitumor, anti-inflammatory, and anticancer purposes, were included. Thus, 88 journal papers were finally included for analysis.

### 2.4. Analysis of the Articles

The data of the 88 journal articles selected for the analysis were tabulated using Bibliometric Scientific mapping analysis and Microsoft Excel. The collected data included the author’s affiliation, year of publication, journal name, and publisher’s name. Analysis was then undertaken of the biodegradable polymer nanocomposites and other natural polymeric biomaterials for tissue engineering and drug carrier applications.

## 3. Results and Discussions

### 3.1. Publication Trends

In this section, we discuss the publication of articles based on the articles’ year of publication, journal, publisher, geographic location of the conducted study, and author affiliation. 

#### 3.1.1. Year of Publication

**Figure 15:**
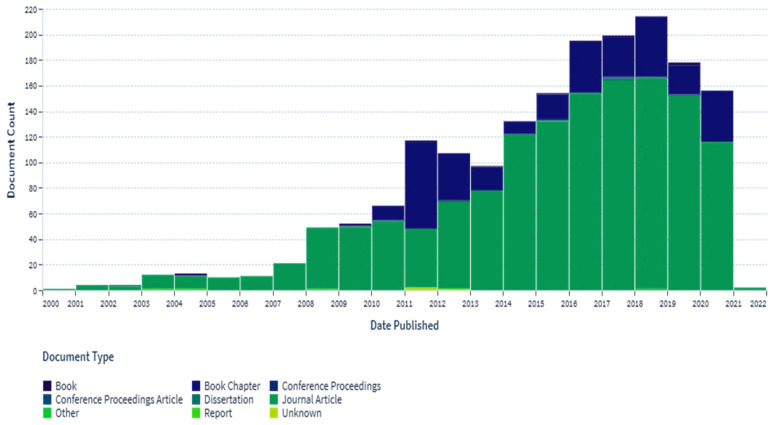
Number of articles published per year.

The articles related to biodegradable and natural polymers for biomedical applications published between 2000 and 2021 were taken into consideration. Here, we consider only the publication trend of the relevant publications that were finally included according to the inclusion and exclusion criteria. The annual publication of the articles is shown in Figure 15. 

It can be noted that researchers increased the study of performance characteristics of polymer carriers for drug delivery in tissue engineering, antimicrobial, antitumor, anti-inflammatory, and anticancer applications in 2014. The lowest number of relevant publications was found in the initial years, i.e., between 2000 and 2013. However, from 2014, a noticeable increase in the number of articles can be seen, with articles numbering 214, 199, 195, and 178 in 2018, 2017, 2016, and 2019 respectively. In 2020, 156 relevant articles were published. 

#### 3.1.2. Journal and Publisher

**Figure 16:**
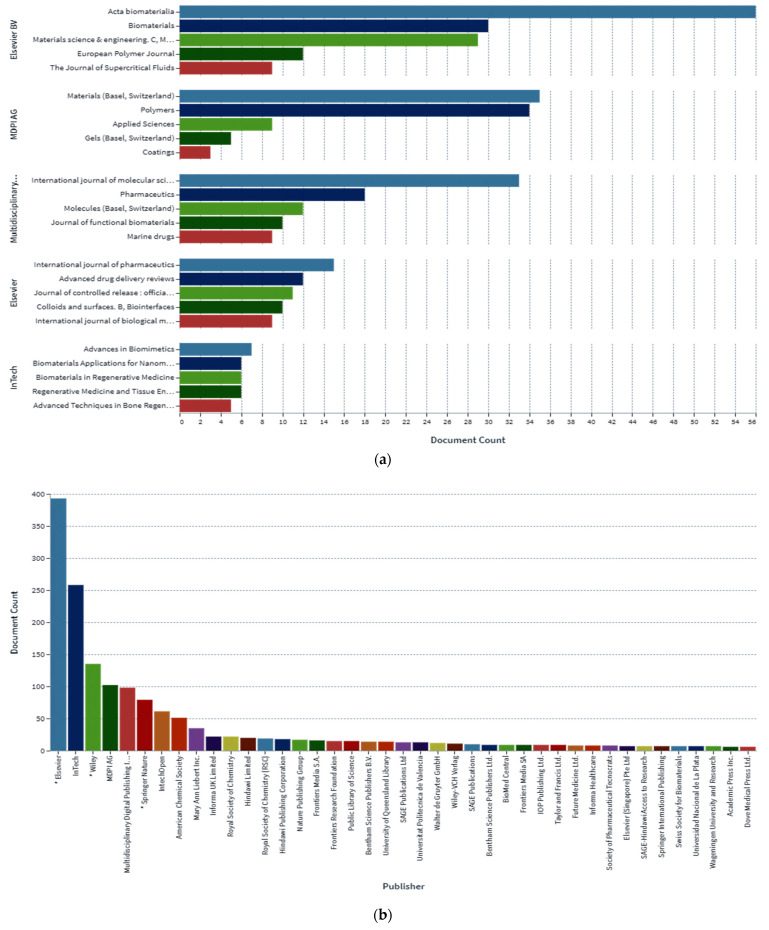
(**a**) Number of relevant articles (*n* = 88) by journal. (**b**) Number of relevant journal articles (*n* = 88) by publisher.

This section shows the classification of relevant published journal articles (*n* = 88) according to the journal and publisher. There are wide varieties of journals and publishers with which researchers were associated for the publication of their studies. A total of 25 journals and 41 publishers were reported during the data processing of the 88 identified articles, as shown in Figure 16a,b. From Figure 16a, it can be seen that *Acta Biomaterialia* was the most popular journals for publishing articles related to the performance characteristics of natural polymeric and biodegradable polymers for tissue engineering and drug delivery applications, i.e., 56 of the total number of articles, followed by *Materials* (Basel, Switzerland) with 35 articles, and the *International Journal of Molecular Sciences*, with 33 articles. Figure 16b shows that Elsevier was the most frequent publisher, contributing to 21.1167%, of the articles followed by Intech (13.86%) and Wiley (7.25%). 

#### 3.1.3. Author’s Affiliation

**Figure 17:**
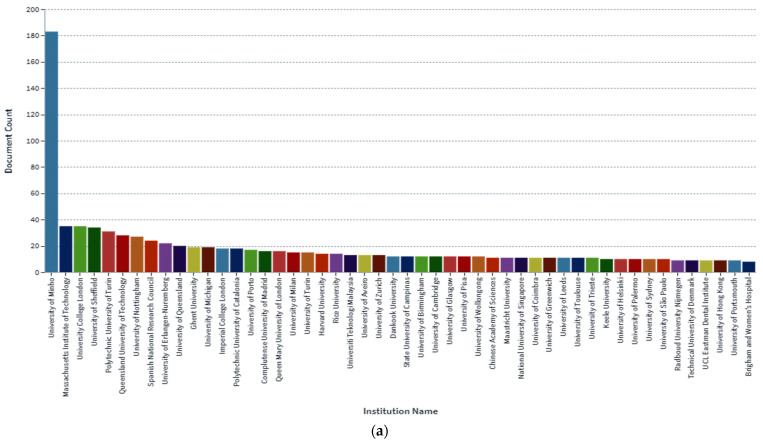

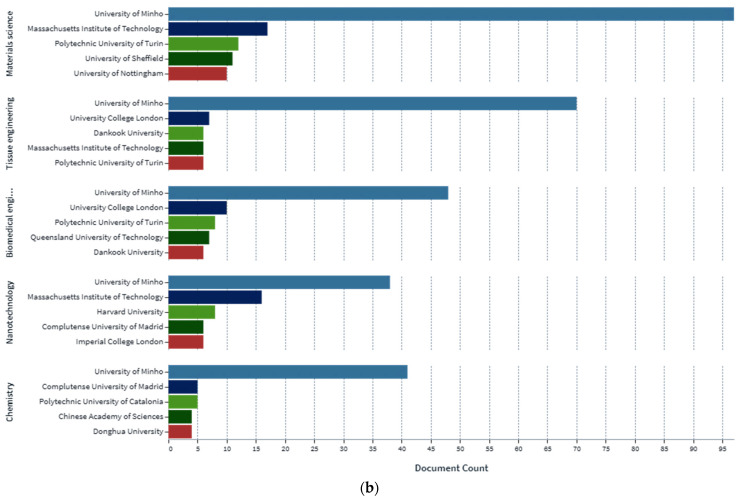
(**a**) Number of papers published (*n* = 88) according to the author’s institute. (**b**) Number of papers published (*n* = 88) according to the author’s institute and field of study.

It was observed during data processing that authors from various countries were interested in the field of biomimetic polymers for biomedical applications. The database used for the present study only gathered the information relating to articles published in the English language. This indicates that the majority of the analyzed articles originated in countries where English language is primarily used for research and technical reports. Thus, the majority of articles was found to come from the UK, followed by Portugal. The distribution of authors, participating institutes, and countries may vary if articles of other languages are considered. The most active institutions in the fields of study related to the identified articles were the University of Minh, followed by Massachusetts Institute of Technology and Polytechnic University of Turin, as illustrated in Figure 17a,b.

**Figure 18:**
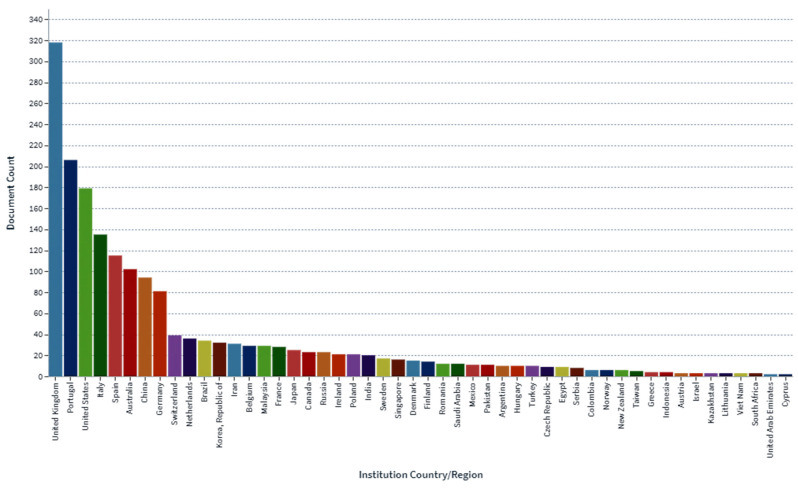
Number of papers published (*n* = 88) according to the geographical location and country affiliation.

The data were tabulated to classify the articles according to the author’s affiliation, as shown in Figure 18. It was found that authors from 50 different institutes or universities, and a variety of countries, undertook the studies reported in these 88 articles. The maximum numbers of authors related to the published articles were affiliated with UK (17%), followed by Portugal (11%) and USA (9.7%).

Following the categorization of the articles according to the author’s affiliation, we report the distribution of articles according to the country in which the experiments were carried out, as shown in Figure 18. In some cases, it was found that studies involved as many as six or seven authors in a single research paper, and affiliations with two or three countries. Figure 18 shows the distribution of the articles according to the country in which the experiments were conducted. Experiments were also carried out in China, Spain, Australia, Malaysia, USA, Mexico, Oman, China, Philippines, Colombia, Portugal, France, and Indonesia.

**Figure 19:**
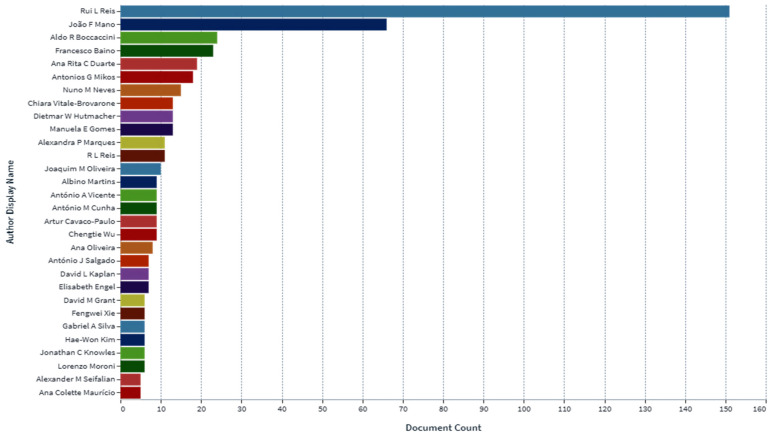
Number of papers published (*n* = 88) according to the most active authors.

The most prolific and dominant author, Rui L. Reis, has the maximum number of articles published, contributing to 30% of the articles, followed by the João F. Mano (13%) and Aldo R. Boccaccini (4.8%), as exhibited in Figure 19.

### 3.2. Drug Delivery Systems of Biodegradable and Other Natural Polymeric Biomaterials in Hard Tissue Engineering

In recent years, a new generation of system has appeared as an alternative solution to many cases of trauma or diseases of bone tissue, in which a wide range of synthetic bone substitutes and biomaterials are used as scaffolds (such as chitosan, alginate, collagen, and hydroxyapatite) [315,316]. The following characteristics are required for the successful implementation of a bone scaffold: It must be able to be sterilized;

- It must provide mechanical support;
- It must deliver bioactive molecules;
- It must not cause inflammatory reactions;
- It must have interconnected pores to facilitate the growth of a new bone;
- It must to promote the osteogenic differentiation;
- It must degrade as the new bone forms;
- It must not create non-toxic degradation products;
- It must sustain the bone cell migration [5].

**Figure 20:**
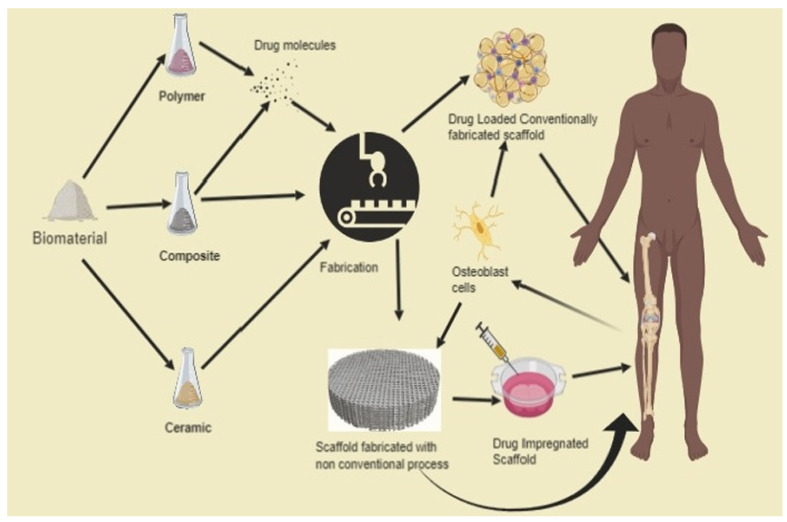
The method in which the scaffold is immersed in the drug solution. Reproduced with permission from [1].

Several methods have been reported for impregnating the scaffolds with drugs [1]. The first method is the simplest, and entails the immersion of the scaffold (with absorbing properties) into the drug solution, as shown in Figure 20. 

**Figure 21:**
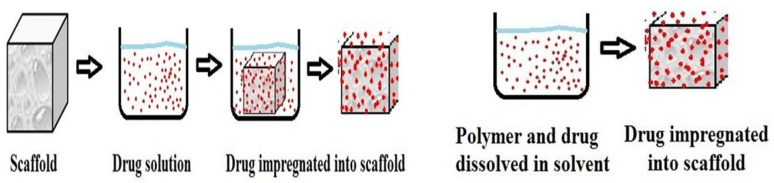
The formation of the system during fabrication. Reproduced with permission from [1].

The second method refers to building the system by dissolving the polymer and the drug in solvent during fabrication, as show in Figure 21. It was reported in the literature that, in the case of these two methods, the drug release profiles depend on the scaffold features, such as degradation rate and porosity [1].

**Figure 22:**
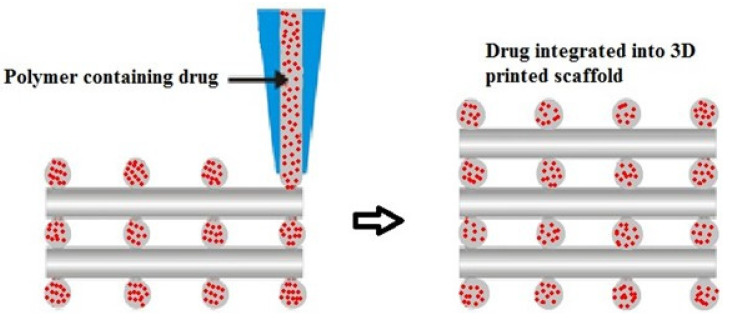
The 3D printing method. Reproduced with permission from [1].

One new method that has revolutionized medicine is 3D printing. The biggest advantage of this method is the accurate control of the architecture, shape, size, location, and dosage of drugs, as shown in Figure 22 [1].

**Figure 23:**
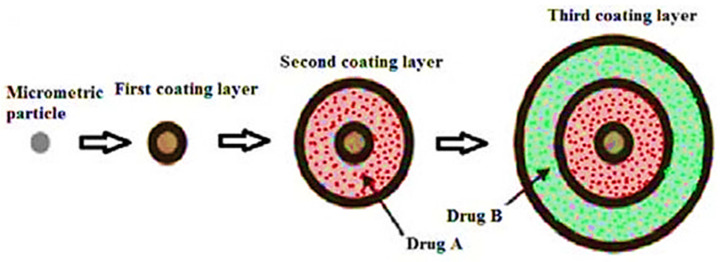
Layer-by-layer method. Reproduced with permission from [1].

Another widely used method to address the side effects of orthopedic implants is layer-by-layer technology, as exhibited in Figure 23. This method involves covering certain surfaces with different layers, between which the drugs (drugs A and B) are caught. Subsequently, capsules can be formed by decomposition [1].

One of the most common global diseases at present is osteoporosis. Most drugs used to treat this disease have been shown to be ineffective due to their side effects. Thus, to prevent these side effects, drug delivery technologies have been used with the effects of enhancing the release profile, reducing toxicity, and improving the therapeutic effectiveness of the drugs [317]. A number of drug delivery systems used in hard tissue engineering are presented in Table 3. The main antibiotics used in hard tissue applications are gentamicin, ampicillin, penicillin, oxacillin, kanamycin, and methicillin from bone cement. Currently, for this application, the researchers are investigating a new type of scaffold that contains antibiotic-loaded nanoparticles [318].

**Figure 24:**
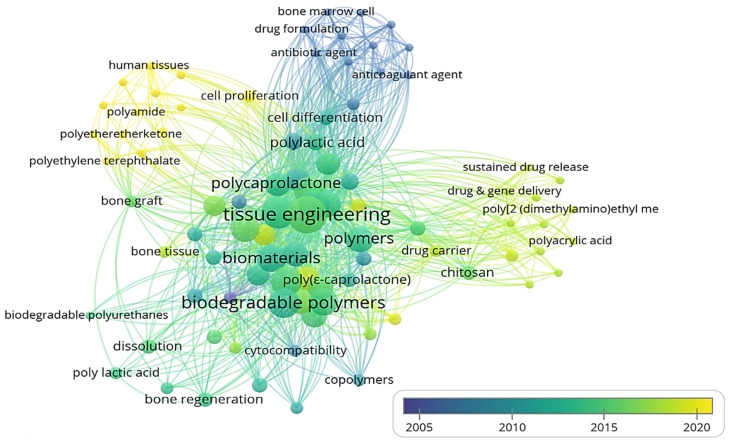
Bibliometric analysis of the utilization of biodegradable and other natural polymeric biomaterials in hard tissue engineering applications.

The bibliometric mapping analysis shows that the use of biodegradable polymers in biomedical applications has undergone significant advances during the past 80 years, as illustrated in Figure 24. Biodegradable materials have emerged for the development of therapy devices, such as provisional implantable devices and 3D scaffolds for tissue engineering. Additional progress has been made in the use of biodegradable polymers for pharmaceutical applications, including drug delivery carriers with sustained delivery. To ensure efficient treatment, these developments require that the materials have desirable mechanical, biochemical, and decomposition characteristics. As a consequence, a wide spectrum of polymeric materials that enable hydrolytic and enzymatic deterioration are now being explored for biological applications, including tissue regeneration, regenerative medicine, prostheses, temporary implants, tissue repair and regeneration, wound healing, and drug carriers.

### 3.3. Polymer Nanocomposites and Natural Polymeric Carriers in Drug Delivery and Biomedical Engineering

**Figure 25:**
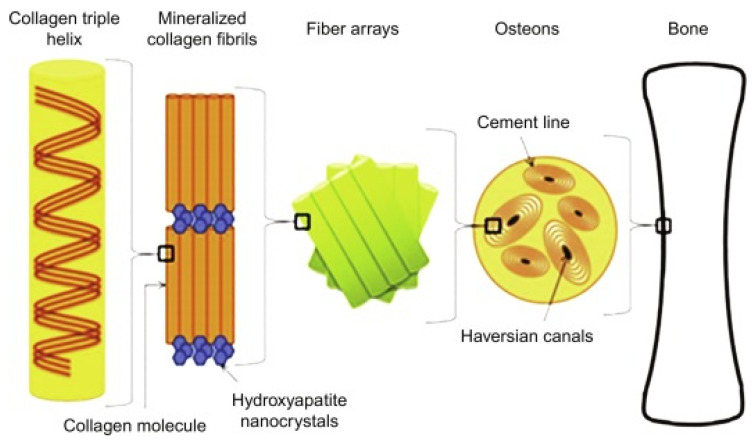
Schematic flow diagram indicating that hierarchical bone arrangement relies on the self-assembly of triple helices of collagen and the accumulation on the surface by HAp-precipitated crystals. The formation of structured and layered fibrous assemblies and osteons (i.e., concentric strands) corresponds to later measures. Reproduced with permission from [319].

Jordi Puiggali et al. (2019) highlighted nanocomposites comprising novel materials. Although nanocomposites have traditionally been developed from natural materials, in this article, the author specifically describes novel materials in the formulation of nanocomposites. Nanocomposites composed of hydroxyapatite, bioactive glass, chitosan, collagen, fibrin, gelatin, and silk were thoroughly investigated. The basic compositions of each of the basic materials, in addition to their synthesis techniques and applications in different fields were reported. Most of the polymers were found to be biodegradable and biocompatible, and to have unique advantages in biomedical and pharmaceutical fields. Important areas within the biomedical field include tissue regeneration and bone implants, whereas key pharmaceutical applications include drug delivery and solubility enhancement, as indicated in Figure 25. The mechanical properties of the above-mentioned polymers were also found to be improved [319].

Alvarez G.S. et al. (2017) discussed the formulation of biomaterial nanocomposites. Biomaterials are agents that are placed in contact with living tissue to improve or replace the unique functioning of the tissue or a particular organ. Tissue engineering has been found to be an important technology in the formulation of functional substitutes to help regenerate and repair damaged tissues or organs. The study also described hydrogels, which are polymers that have an open core structure and retain a large amount of water in their structure. Hydrogels were found to consist of two types, namely, from natural and synthetic polymers. Due to their distinctive properties, such as biocompatibility, natural polymers have wide applications in the biomedical field, and include polymers such as chitosan, collagen, and gelatin. Synthetic nanocomposites composed of polylactic acid, polyglycolic acid, polymethyl methacrylate, and polyvinyl alcohol were also discussed in details [320].

Priscila Anadao et al. (2012) highlighted the concept and applications of polymer-clay nanocomposites. In 1949, Bower conducted an experiment for DNA absorption using montmorillonite clay. In 1963, Greenland highlighted the use of polyvinyl alcohol montmorillonite nanocomposites. Subsequently, a large amount of research has been carried out on polymer-clay nanocomposites. These are composites that have a polymer matrix, whose dispersed phase is formed by silicate particles that have dimensions in the nanometer range. Based on the interphase forces in clay and polymer, the authors highlighted different thermodynamically accepted morphologies. Intercalated, exfoliated, and flocculated morphologies are described for nanocomposites. In addition, methods of preparation of polymer nanoclay nanocomposites were briefly discussed. Important methods include in situ polymerizations, solution dispersions, and fusion intercalations. In in situ polymerization, the clay particles are dispersed in monomer medium and, using suitable conditions, the polymerization process is carried out. The use of a compatibilizing agent is also highlighted in the literature. The name indicates that the agent is compatible with clay and the polymer. An example is maleinanhydride, which is used as a compatibilizer for polypropylene and polyethylene polymer. The widely used polymers for the preparation of nanocomposites are polyethylene, polypropylene, polyvinyl chloride, polyamide, and polyethylene oxide. The use of biodegradable polymer from natural and synthetic sources was also mentioned. These sources include polyhydroxy butarate, chitosan, polycarbolactone, and polylactic glycolic acid. The application of polymer nanoclay nanocomposites was also reported in literature. These nanocomposites can be used in different areas, such as the biomedical applications of artificial tissues, dental and bone surgery, and medicine and drug delivery. Other applications include automatic energy, packaging, and construction [321].

**Figure 26:**
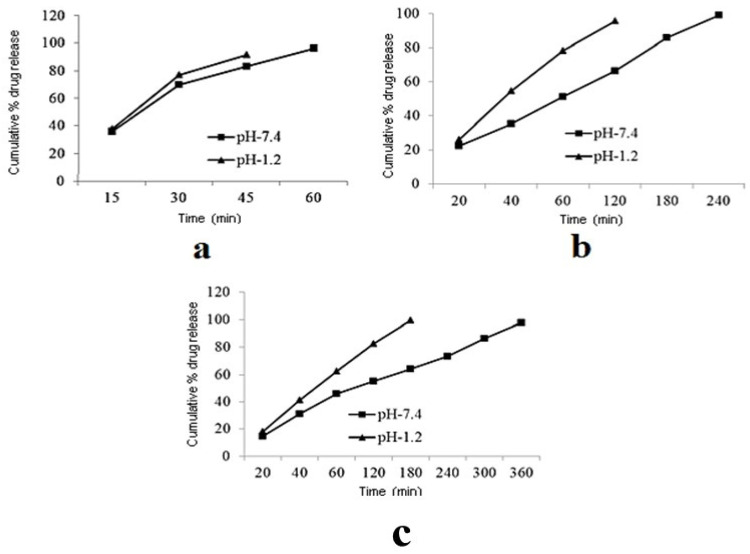
Influence of concentration on the cumulative percentage of drug delivery at (**a**) 0.05%, (**b**) 0.1%, and (**c**) 0.15% of capoten-loaded nanocomposite hydrogels. Reproduced with permission from [322].

S. Latha et al. (2013) formulated hydrogels of Captopril nanocomposites. Due to their better diffusion, biocompatibility, and excellent water sorption, hydrogels have wide applicability in the biomedical field. The authors developed hydrogels of nanocomposites using a novel free radical reaction technique of polymerization based on nanoclays of montmorillonite. The formulated hydrogels were evaluated in terms of their swelling behavior, SEM, DSC, TGA, FTIR, drug loading, and in vitro drug release. It was observed that the pH of the external media and the clay addition order had a profound effect on the swelling characteristics of nanocomposite hydrogels, as shown in Figure 26. The targeted release of Captopril was observed in the intestine [322].

Karak et al. (2019), highlighted the fundamental concept of nanocomposites of polymers and nanomaterials. Nanomaterials are defined as materials whose dimensions are less than 100 nm. They also highlighted how shape, surface structure, and size influence the properties of nanocomposites. The developed nanocomposites have unique thermal, biodegradability, and mechanical properties; in addition, their flame retardant ability was found to be enhanced. The historical perspective, classifications, and raw materials required for the formulation of nanocomposites were discussed in the study. In addition, the methods used to prepare polymer nanocomposites, including physical and chemical approaches, were discussed. The study also highlighted the significant properties of nanocomposites, such as mechanical strength, optical activity, toughness, catalytic activity, thermal stability, biological activity, and barrier properties. The improvement in the properties of nanocomposites with the use of a core polymer was also discussed in detail. The various applications of nanocomposites were outlined in the study; these applications include use in the biomedical, pharmaceutical, industrial, agricultural, sports, and electronics fields [323].

Bhat. M. et al. (2015) reported that the Biopharmaceutical Classification System (BCS) Class II drugs have poor aqueous solubility. This affects the drug’s release, which is significantly influenced by the aqueous surrounding in the gastro-intestinal (GI) tract, which particularly affects the drug’s bioavailability. Bio-nanocomposites are a hybrid form of biopolymer in which two or more components are fused together. These have numerous applications in different dosage forms. The review highlighted the simple and convenient method of preparation of bio-nanocomposites using the microwave irradiation method by means of carriers of natural origin, such as acacia, ghatti gum, cassia, and gelatin, to enhance the solubility of BCS Class II drugs and improve the rate of dissolution for these drug entities, thereby affecting their bioavailability. The reported fusion method (MW) offers numerous advantages, such as its simplicity, time saving, and cost effectiveness. The MW technique is a recent and advanced technology in materials processing and chemical manufacturing, and presents promising advantages compared to the traditional thermal treatments. The mixture was heated to form a molten mass which was cooled and solidified. The final product was crushed and sieved. The developed nanocomposites can be characterized by different analytical techniques, such as SEM, FTIR, DSC, and XRD. At present, materials processed via the MW approach are extensively used in pharmaceutics that are developed specifically to enhance the speed and efficiency of the extraction process of polar solvents [324].

Paul D.R. et al. (2008) synthesized a polymer matrix nanocomposite and suggested it may be used in targeted drug delivery applications. The addition of nanoparticles to the drugs provided significant advantages, and resulted in a slower and steadier drug delivery process that enabled more control of the release, enhanced the mechanical integrity of the hydrogel-based nanocomposites, and reduced swelling. In the literature, iron oxide nanoparticles have been examined for a wide range of applications, such as immunoassays, cellular therapies, magnetic resonance imaging contrast enhancement, and drug delivery. These experimentations usually deploy magnetic dispersal in a polymeric microsphere or microcapsule, involving biodegradable and/or natural polymers of poly(L-lysine) microspheres containing magnetic nanoparticles. These were manufactured through coacervation and were characterized for further potential use, such as in targeted drug delivery systems. The Fe and Co nanoparticles encapsulated in polydimethylsiloxane were tested for the treatment of retinal detachment disorder [325].

Kushare S. et al. (2013) synthesized bio-nanocomposites using microwaves to improve the dissolution and solubility of the poorly water-soluble drug, Glipizide. However, when the material was characterized, it was confirmed that there no interactions occurred between the polymers and the drug. The researchers then concluded that Glipizide was converted into nanocrystals in the composites, to which the improvement in solubility was solely attributed. The use of microwave irradiation generated by a microwave oven caused the breakage of the internal structure of the drug particles, resulting in the formation of nanoparticles, and ultimately leading to an enhancement in solubility. The in vitro and in vivo evaluations conducted for the optimized formulation reaffirmed the use of BNCs to improve the dissolution and solubility using natural carriers. In stability studies conducted by the researchers, the BNC-containing formulations were found to be stable. The microwave irradiation method is a novel method for the improvement in drug solubility [326].

**Figure 27:**
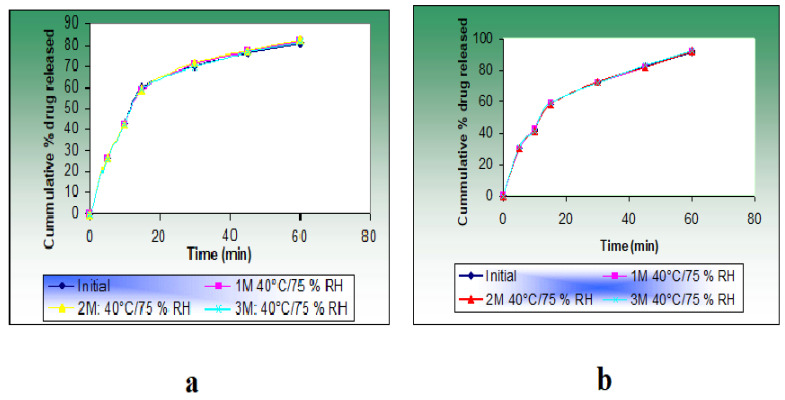
Comparison of in vitro dissolution characteristics of Meloxicam/Poloxamer solid dispersions fabricated using (**a**) the melting process and (**b**) the microwave method. Reproduced with permission from [327].

Mukhija Umesh et al. (2012) emphasized the challenge of the poor aqueous solubility of drugs in their formulation. They also stressed that the drug must be eventuality converted into a bio-nanocomposite using natural carriers. The attempts to enhance the solubility rate of Meloxicam were reported because this drug has poor water solubility, and its solubility–dissolution was improved using different solid dispersion techniques based on Poloxamer 188. The solid dispersions of Meloxicam for in vitro dissolution were prepared by two methods, namely, the microwave-assisted method and the hot melt method. From the results obtained from the formulation, it was found that the model with the best fit for the mechanism of dissolution was the Higuchi matrix release, which had the highest release. When compared with the melting method, the microwave-assisted method proved to be highly compatible for producing better solubility in the preparation of a solid dispersion, as exhibited in Figure 27. Microwave irradiation is an effective and efficient instrument in the generation of molecular dispersion [327].

D. Bikiaris et al. (2008) synthesized a solid dispersion of Tibolone from polyethylene glycol to increase the dissolution rate of drugs with poor water solubility using microwave irradiation. The researchers found that the time required for dissolution of Tibolone in PEG melting under microwave irradiation was significantly less than that of the traditional melting method using heat application (>15 min). Thus, they determined that microwave-induced synthesis of solid drug dispersions is a simple and efficient process compared to traditional methods of preparing solid dispersions, such as melt mixing or solvent evaporation through heat application. The application of microwaves at the instance at which Tibolone solid dispersions were synthesized in PEG resulted in the formation of various-sized drug crystals in the dispersion, compared to the solid dispersions manufactured using traditional methods. Furthermore, the researchers also noted and compared the results obtained in terms of various drug properties. It was mentioned that the drug’s dissolution rate appeared to be higher when the dispersions were formulated using microwave irradiation [328].

M. Moneghini et al. (2008) synthesized solid dispersions of ibuprofen using microwaves. For this purpose, the active system was prepared with PVP/VA/60/40, whereas HP-Cyclodextrin was designated as the carrier. This significantly increased the dissolution profile of ibuprofen, which is usually considered to be a poorly soluble drug. Furthermore, the researchers analyzed the physical characteristics of the microwave-activated system. They found that the drug was completely amorphized and no polymorphic forms were found. This study deployed the microwave technique to prepare SD. The study results indicated that this is a viable technique and a suitable alternative in the preparation of solvent-free binary systems. In contrast to the conventional heating process, in which heating is confined to the surface, heating produced by this technique is uniform across the material. Unwanted side reactions are also mitigated (reaction quenching). In this study, the active system was prepared with PVP/VA 60/40, whereas HP-Cyclodextrin was designated as the carrier [329].

**Figure 28:**
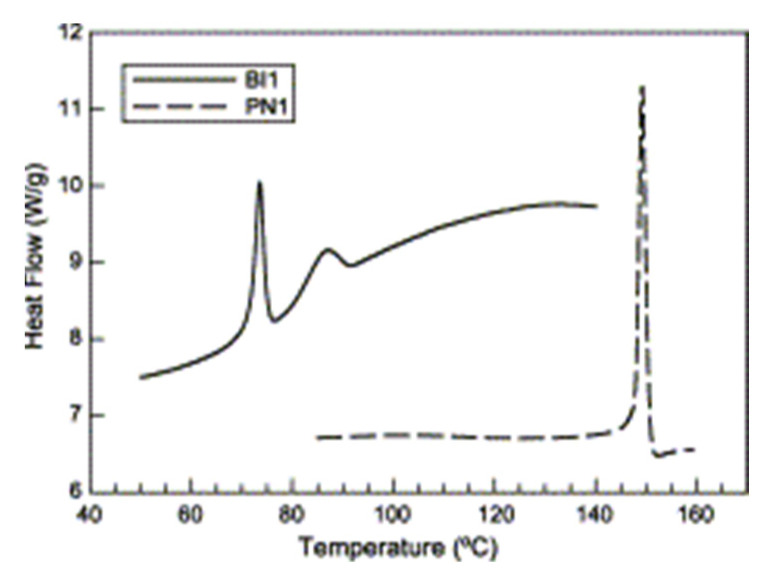
DSC thermogram. Solid line: Composite BI1; the 75 °C peak conforms to the melting point of the microcrystalline ibuprofen stage, whereas the β-CD dehydration is correlated with the highest point at 85 °C. Broken/dotted line: Composite PN1; the maximum point is correlated with the melting of the microcrystalline nimesulide stage. Reproduced with permission from [330].

P. Bergese et al. (2003) synthesized nanocomposites using microwaves to increase the solubility of drugs. Microwave irradiation is a widely used technique that offers numerous advantages compared to traditional thermal processing. Ibuprofen, nimesulide, and nifedipine were the model drugs utilized from BCS Class II, whereas polyvinyl pyrrolidone and βCyclodextrin were the polymers used. When characterized, the materials confirmed that there was no interaction between the polymer and the drug. The study concluded that microwave processing has a positive and significant impact on the drug, i.e., transformation from a microcrystal to a (matrix embedded) molecular cluster, as shown in Figure 28 [330]. Such (thermally activated endothermic) transition, as confirmed by DSC & Thermo-gravimetric analyses (Figure 3), seems to be a solid/solid shift transformation in which the hydrates β-CD ends up losing the water of crystallisation (de-hydration).

D. Maurya et al. (2010) synthesized poorly water-soluble atorvastatin calcium using microwaves, which induced its solubility. Their solid dispersions of atorvastatin and PEG 6000, generated using the microwave-induced fusion method, notably enhanced the rate of dissolution (*p* < 0.05). The researchers found that the reasons for the improved dissolution rate were the solubilizing effect of PEG 6000, increased wetting, alteration of the drug surface properties, and the molecular dispersion of the drug in solid dispersions. When the material was characterized, the researchers confirmed that there was no interaction between the polymers and the drug. They also concluded that the results of solid dispersions prepared by the MIND method under in vivo conditions showed increased solubility [331].

**Figure 29:**
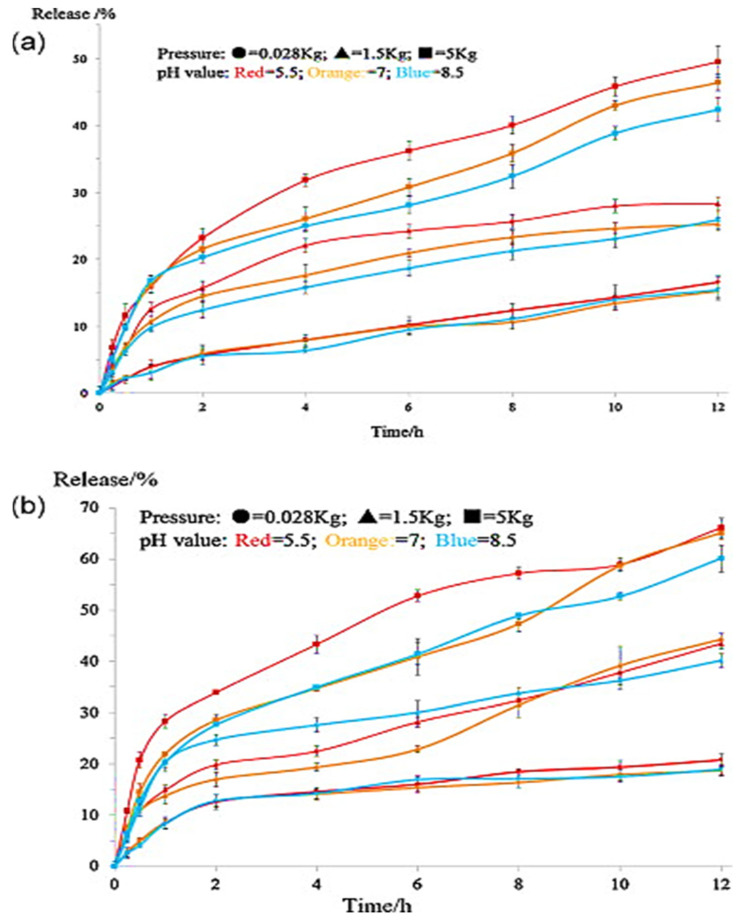
Quantity of (**a**) chitosan/miconazole nitrate and (**b**) chitosan/clotrimazole microspheres released in vitro from the drug at diverse pressures at various concentrations. Reproduced with permission from [332].

Yuen M. et al. (2017) synthesized an advanced microcapsule that could be further applied via bandages or socks to release antifungal drugs to treat fungal skin diseases in a controller manner under pressure, as shown in Figure 29. Chitosan/miconazole nitrate and chitosan/clotrimazole microcapsules were the two kinds of microcapsules prepared for the study. The mean particle size was 2.6 µm for the chitosan/miconazole nitrate microcapsules and 4.1 µm for the chitosan/clotrimazole microcapsules. High-performance liquid chromatography (HPLC) was used to determine the drug loading and encapsulation efficiency. The above-prepared microcapsules, loaded with the drug, can be directly applied to socks or bandages that release antifungal drugs in a controlled manner under pressure. Patients who suffer from tinea pedis or other fungal infections can administer this medical treatment by wrapping a bandage around the body area or putting on socks [332].

**Figure 30:**
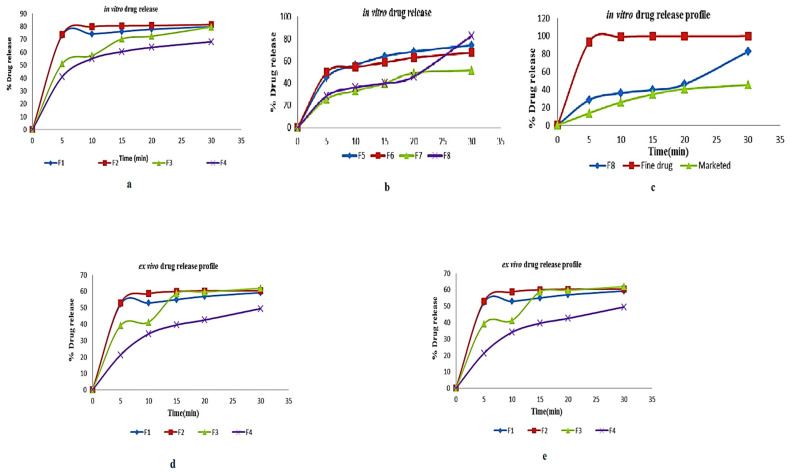
(**a**,**b**) In vitro drug release of synthesized hydrophilic polymer–gel in various formulations; (**c**) in vitro drug release of synthesized hydrophilic polymer–gel in the marketed product formulation; and (**d**,**e**) ex vivo drug release profile of developed gels in different formulations. Reproduced with permission from [333].

Salomy M. et al. (2015) created a topical gel for application to the skin or specific mucosal surfaces. The aim of the gel was to perform local actions or transdermal penetration of the medicament, or for its emollient or protective actions. The study attempted topical delivery of the drugs directly upon the hydrogel matrix, so that the drugs were effectively delivered at the required site and, simultaneously, avoided first pass metabolism, enhanced local action in pain management, and treated skin diseases. Hydrophilic polymers, such as guar gum and Carbopol 940, of varying concentrations, were used to develop a topical hydrogel formulation of the drugs, as exhibited in Figure 30a–e [333].

Akhilesh K. Gaharwar et al. (2014) specifically examined the updated information on nanocomposite hydrogels, with a particular focus on biomedical and pharmaceutical applications. These are hybrid hydrogels having a hydrated network of polymers that are cross-linked with each other or nanostructures. The researchers highlighted the advances in the field of nanocomposite hydrogels in terms of their physical properties and applications. Two-phase and multi-phase systems were also discussed. Due to their porous and hydrated molecular structure, the nanocomposite hydrogels usually stimulate the native tissue microenvironment. The factors and challenges associated with the fabrication and design of nanocomposite hydrogels were also discussed. This study provided a novel approach to reinforcing polymeric hydrogels, including different functionalities that were focused on the implementation of nanoparticles within the hydrogel network [334].

**Figure 31:**
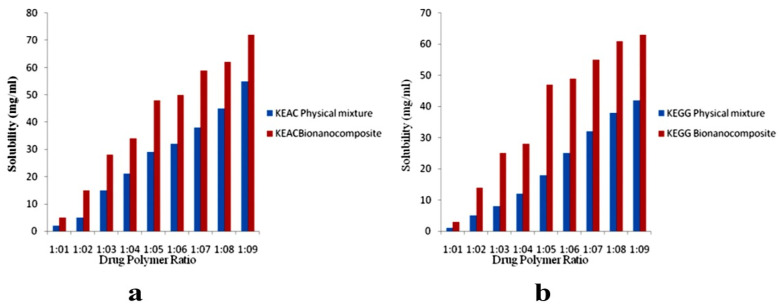
(**a**) Comparison of the solubility between the physical KEAC combination and KENC. (**b**) Correlations of solubility in KEGG physical blend and KEGG bio-nanocomposite. The information is the average ± SD, *n* = 3. The results are expressed in terms of the solubility percentage of virgin ketoprofen, where: KE–ketoprofen, AC–acacia, GG–ghatti gum, SD–standard deviation. Reproduced with permission from [335].

Surendra G. Gattani et al. (2016) developed bio-nanocomposites using the microwave-induced diffusion technique (MIND) to enhance the solubility of the drug ketoprofen. They highlighted the importance of solubility in achieving the concentration of the drug in the systemic circulation. Among all of the available drug molecules, only 8% have sufficient solubility. Different formulations were developed using the microwave-induced diffusion technique. The MIND process is an effective and simple technique for the enhancement of the solubility of molecules. It is an advanced and current technology in materials processing and the manufacture of chemicals, and presents promising advantages compared to the traditional thermal treatments. Heating is a significant component in the energy exchange. The solubility enhancement of bio-nanocomposites was investigated by dissolution and an in vitro solubility study, as shown in Figure 31. The polymers were selected based on the surfactant and the wetting properties. The solubility of ketoprofen was enhanced using the microwave-induced diffusion technique. The microwave technique enables rapid and uniform heating of materials with low heat conductivity because energy can easily be converted into heat within the material and most of the materials comprise polymers. The enhancement in solubility may be attributed to the drug dispersion at micro- and nanoscales. The in vivo study of optimized bio-nanocomposites was also conducted using the rat paw edema model [335].

**Figure 32:**
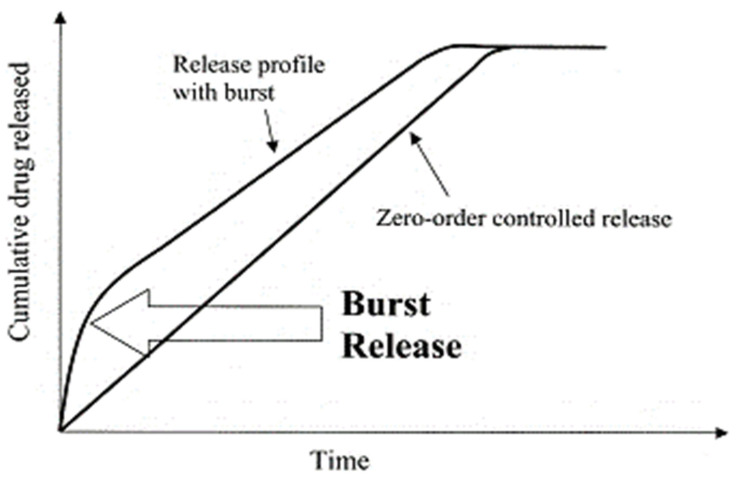
Schematic representing the bursting effect in a zero-ordered drug delivery system. Reproduced with permission from [336].

Xiao H. et.al. (2000) analyzed the mechanisms and the important role of the burst release in drug delivery systems under the control of the matrix. In this research, the authors reviewed the burst release experiments on monolithic polymer-controlled drug delivery systems. Furthermore, they reviewed the theories regarding the physical mechanisms that cause bursting, and presented novel ideas to prevent bursting and to treat burst release under controlled release models. This article also discussed the significance of burst release and suggested that burst release may be applied in the treatment of wound and bacterial bone infection, in which an initially high concentration of antibiotics is needed for effective eradication of the infection, as illustrated in Figure 32. The burst release profile is also useful in the case of targeted drug delivery and pulsatile drug delivery systems [336].

**Figure 33:**
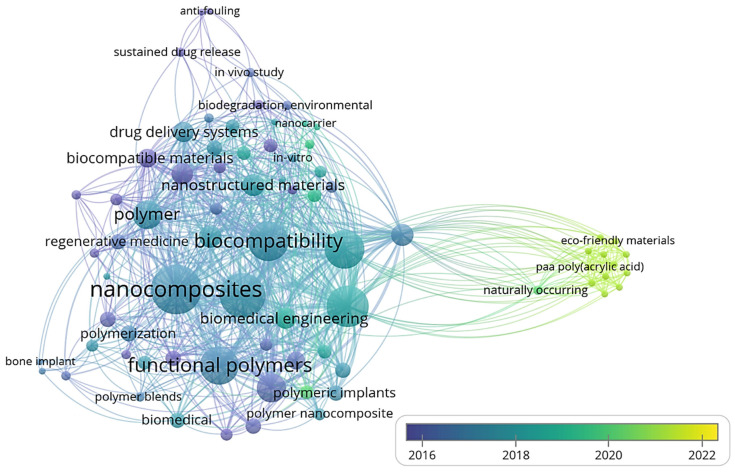
Bibliometric analysis of the utilization of natural polymeric biomaterials and nanopolymers in drug carriers and tissue engineering applications.

Nanostructured polymers have attracted increased attention as a promising class with functional materials, and in the design of biocompatible frameworks for biomedical applications, as shown in the bibliometric mapping analysis in Figure 33. The combined effect of multiple classes of nanomaterials enhances not only the inherent characteristics of composite materials, but also their shape, and their stereochemical, biological, functional, and compositional resemblance to organic and inorganic body parts. A broad spectrum of formulations, mixtures, and nanofillers is comprehensively used in medical applications, primarily in the role of a drug carrier. The key obstacle for polymeric nanocomposites is to mimic (biologically, synthetically, and functionally) the extracellular matrices of numerous body parts to facilitate tissue regeneration. 

### 3.4. Antimicrobial Drug Delivery of Biodegradable and Other Natural Polymeric Biomaterials in Hard Tissue Engineering

Antibiotics are frequently used in the case of bone implants to prevent postsurgical infection or in the case when an infection has been diagnosed [3]. For example, in the case of osteomyelitis, the classic treatment entails the surgical removal of the diseased bone followed by the administration of antibiotics. This method is complicated because it causes weakening of the musculoskeletal support and the effectiveness of the antibiotics decreases. Thus, this problem can be resolved using systems capable of local delivery of antimicrobial agents [2]. Gomes D. and co-workers reported that composite nanostructures, such as hydroxyapatite and PLGA, are used for the treatment of osteomyelitis and the delivery of antibiotics to the infected bone [337]. Examples of drug delivery systems for osteomyelitis are presented in Table 4.

Logith Kumar R and co-workers reported that the antimicrobial activity of chitosan can be improved by modifying its structure, thereby increasing its suitability for use in hard tissue engineering [338]. In addition, the team of researchers investigated the antimicrobial activity of the incorporated vancomycin-loaded liposomes into a nano-hydroxyapatite-chitosan-konjac glucomannan scaffold. 

It was reported that the scaffold was biocompatible and biodegradable, and enabled modification of the release profile of the drug by adjusting the ratio between the chitosan and konjac glucomannan. The study used a scaffold with a content of 60–70% nano-hydroxyapatite, and the content of chitosan and konjac glucomannan was varied to allow the differences to be observed. A slower release of the drug was observed at the largest amounts of chitosan and konjac glucomannan used. The in vitro tests confirmed that the system comprising vancomycin-loaded liposomes and scaffolds resulted in greater inhibition of the formation of *S. aureus* biofilms than the drug-loaded scaffold [339].

In another study, Hornyák I and co-workers studied the antimicrobial activity of human bone allografts incubated with antibiotic solution (vancomycin) and coated with chitosan. Sustained release of the drug for 50 days was observed. It was reported that the MIC for Enterococcus faecalis was 0.2 µg/mL of vancomycin, and for methicillin-resistant S. aureus, was 2 µg/mL of vancomycin [340]. Another advantage is that the alginate and allograft are biodegradable, which makes the development of the biofilm more difficult [341]. Another study reported on a system formed from a chitosan scaffold with bactericidal agents coated with a nano-hydroxyapatite-poly(amide). The study reported the continued release of the bactericidal agents for over 150 h, the decrease in the extent of bacterial growth, and cell adhesion. In addition, it was reported that scaffolds made of chitosan/nano-hydroxyapatite/nano-silver particles showed good antimicrobial activity against Gram-negative and Gram-positive bacterial strains. Furthermore, it was observed that these scaffolds are not toxic to rat osteoprogenitor cells or human osteosarcoma cell lines [342]. 

González-Sánchez MI and co-workers attempted to maximize the antimicrobial activity of osteoconductive acrylate hydrogels against *Staphylococcus epidermidis* and methicillin-resistant *Staphylococcus aureus* by charging silver nanoparticles using three methods. The first method encapsulated the silver nanoparticles during the synthesis. It was observed that the hydrogels with different cross-linking degrees containing silver nanoparticles showed no changes in antimicrobial activity compared with the control (Ag 0%) against *Staphylococcus epidermidis* and methicillin-resistant *Staphylococcus aureus*. The second method diffused the nanoparticles into the composite by diluting the sodium dihydrogen phosphate in the silver nanoparticle suspension. A slightly higher antibacterial activity was observed compared to the control, but it was reported that these results were not statistically significant. The third method used the adsorption of silver nanoparticles into the scaffold by placing the silver nanoparticle suspension in contact with the mineralized hydrogel for a period of between 1 and 6 days. It was observed that the samples that were in contact with the 1 mM silver nanoparticle suspension showed significantly higher antimicrobial activity compared to the samples that were in contact with the 0.5 mM silver nanoparticle suspension, against both *Staphylococcus epidermidis* and methicillin-resistant *Staphylococcus aureus*. In addition, it was reported that the greatest antimicrobial activity of the scaffolds was achieved for *Staphylococcus epidermidis* and, for the samples that were in contact with silver nanoparticles for 2 days, the antimicrobial activity decreased thereafter. It was reported that this method does not have a negative impact on osteoblasts. This is one of the few studies performed on the acrylate hydrogel with antimicrobial activity using a non-antibiotic-based antibacterial [343].

**Figure 34:**
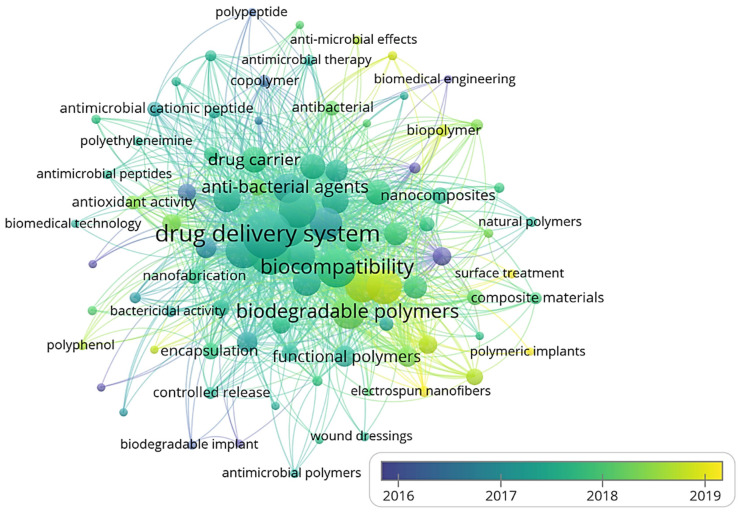
Bibliometric mapping of the antimicrobial drug delivery of biodegradable and natural polymeric biomaterials in hard tissue engineering.

Polymeric biomaterials have had a substantial influence for a sustained period. During the past several centuries, biomimetic and biodegradable polymeric materials have emerged, and have promised exceptional advances in a diverse variety of diagnostic and therapeutic medical devices. Awareness of the interfacial interrelations of polymeric biomaterials, and controlling these materials with biological components such as water, ions, peptides, enzymes, microorganisms, microbes, and cell types, appears to be crucial for their productive use in biomedical fields. This is shown in the bibliometric mapping analysis in Figure 34. 

### 3.5. Antitumor Drug Delivery of Biodegradable and Other Natural Polymeric Biomaterials in Hard Tissue Engineering

Gu W and co-workers reported that the skeleton is the organ that has the highest mortality percentage and is most affected by metastatic cancer [344]. To overcome the limitations of chemotherapy (nonspecific biodistribution and targeting) in the case of cancer, research attention is rapidly focusing on drug delivery systems [345]. For example, El-Kady and co-workers synthetized lithium-modified bioactive glass nanoparticles using the sol-gel method, in which the nanoparticles were loaded with 5-fluorouracil. The release profile of the drug was in two phases: rapid release in the first 24 h, followed by slow release for 32 days. It was reported that the in vitro bioactivity assessment in SBF indicated that this system can be used for bone engineering, and that the controlled release of lithium ions accelerates bone regeneration [346]. In another study, mesoporous silica nanoparticles were synthesized with a dimension of 40 nm, anchored by zoledronic acid, and loaded with doxorubicin for bone cancer therapy. It was reported that the system had improved bone-targeting ability compared with the mesoporous silica nanoparticles. Although the mesoporous silica nanoparticles presented a maximum loading capacity of 1671 mg/g and a loading efficiency of 83.56%, compared to the DOX@MSNs4ZOL system, which presented a maximum loading capacity of 1547 mg/g and a loading efficiency of 77.34%, it was reported that DOX@MSNs4ZOL offered better cytotoxicity against A549 cells and decreased cell migration in vitro [347]. 

**Figure 35:**
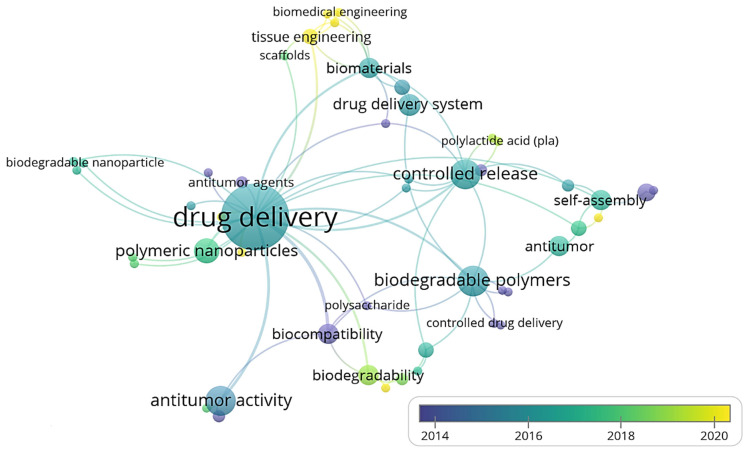
Antitumor drug delivery of biodegradable and natural polymeric biomaterials in hard tissue engineering.

Nanotech advances have contributed to the emergence of novel polymeric compositions that enable the modulation of the biotech and biomedical characteristic rates of compounds. The unique physicochemical and technical attributes of polymeric nanocomposite-based therapeutic agents have resulted in numerous promising therapeutic applications. The utilization of polymer–nanomaterials as anti-cancer compound drug carriers, their physical characteristics, and their ability to be effectively concentrated in particular tumors, were portrayed in this review. The nano-encapsulation of antitumor productive substances in biocompatible polymers is a viable strategy for increasing the effectiveness of numerous tumor treatment options, as depicted in the bibliometric mapping analysis in Figure 35.

Yang L. and co-workers reported that selenium nanoparticles present biocompatibility and anticancer activities, and, when grown on titanium, have the ability to inhibit the growth of cancerous osteoblasts and increase the growth of healthy osteoblasts [348]. Another study investigated new drug delivery systems formed from calcium phosphate cement, calcium phosphate cement containing caffeine or cisplatin, and solely caffeine and cisplatin. The in vitro tests on SOSN2 cells demonstrated that the system formed from calcium phosphate cement, caffeine, and cisplatin released a greater quantity of the drug. In addition, in vivo tests on male Fischer 344/NSlc 7 week old rats demonstrated greater tumor growth inhibition when the calcium phosphate cement, caffeine, and cisplatin system was used. Based on these studies, the authors reported that this system possesses suitable antitumor effects [349]. 

**Figure 36:**
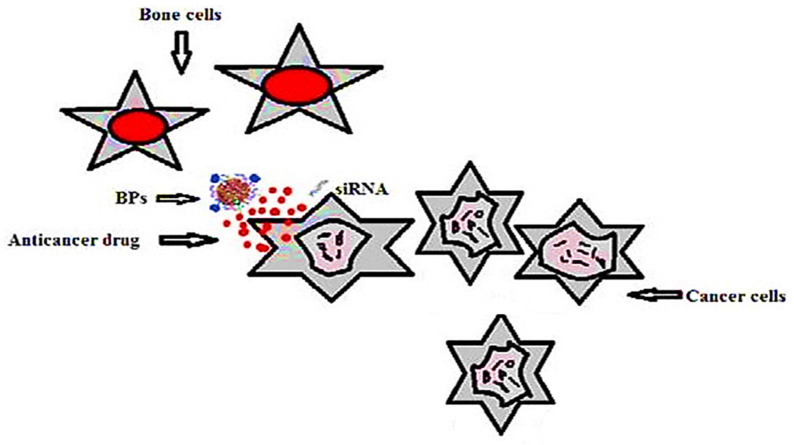
Drug delivery system for bone cancer.

A new class that treats cancer bone metastasis is represented by the bisphosphonates, which show an affinity for bone tissue and can be used to deliver other anticancer drugs. Figure 36 presents a drug delivery system for bone cancer. The system involves mesoporous silica nanoparticles loaded with anticancer drugs and coated with bisphosphonates. The positive charge of the nanoparticles can be transported with siRNAs. At the moment of administration, the nanoparticles remain attached to the bone cells, kill the cancer cells, and release drugs or siRNAs. It was reported that poly-l-lysine grafted with beta-cyclodextrin for RIS delivery warned of the induction of metastatic cancer in animal models [344]. 

Wang F and co-workers created a liposomal system conjugated with cyclic arginine-glycine-aspartic acid-tyrosine-lysine peptide (cRGDyk)-loaded cisplatin. It was reported, after in vivo tests, that this system presents low organ toxicity and high therapeutic efficacy, and can be successfully used for therapy of bone metastases [350]. Another study developed an anti-tumoral-loaded bone graft material for the treatment of bone cancer. The system consisted of collagen, hydroxyapatite, and cisplatin, and was tested on the osteosarcoma G292 cell line. It was observed that the cytotoxic, anti-proliferative, and anti-invasive activities depend on the released concentration of cisplatin [351]. 

Hyperthermia can be used for the destruction of cancer cells. For this purpose, magnetic nanoparticles are loaded onto the scaffold and exposed to alternating magnetic fields, in combination with anticancer drugs. This method is widely used in hard tissue engineering [1]. In addition, Zhang and co-workers created a scaffold consisting of Fe_3_O_4_ nanoparticles, mesoporous bioactive glass, and polycaprolactone produced using the 3D printing technique. It was reported that this system presents excellent apatite-forming bioactivity, good magnetic heating properties, and can be used for the treatment of bone tumors [352].

### 3.6. Anti-Inflammatory Drug Delivery of Biodegradable and Other Natural Polymeric Biomaterials in Hard Tissue Engineering

Conventional nanocarriers have been replaced by nanotherapeutics due to their advantages, such as simultaneous delivery of multiple drugs and the presence of the targeting agents on the surface. These systems can be used to treat different pathologies, such as inflammatory diseases, and can be adjusted depending on the patient. It was reported that chitin dressings accelerate wound repair and can regulate the secretion of inflammatory mediators, such as prostaglandin E, IL-8, and IL-1 β [353]. 

In the case of anti-inflammatory applications, steroids and non-steroids (ibuprofen) are commonly used. For example, Paris and co-workers created a scaffold consisting of apatite and agarose polymer loaded with two drugs (ibuprofen and zoledronic acid) during the scaffold fabrication and after consolidation. In the first step, the agarose polymer was introduced into deionized water and was subjected to magnetic stirring under heating to 90 °C. Then, the temperature was gradually reduced to 45 °C, and the apatite and drug 1 was added. The scaffold was then shaped and freeze dried, and drug 2 was injected into the scaffold. It was observed that this system provides a very fast delivery of ibuprofen (to reduce the inflammation after implantation) and zoledronic acid (to promote bone regeneration). Due to its rapid release, the authors encapsulated the ibuprofen into chitosan spheres. It was reported that, as a result of this change, a release profile was obtained that is suitable for clinical application [354]. Another study developed an anti-inflammatory delivery system for bone applications formed from porous β-TCP pellets loaded with ibuprofen by physisorption. It was reported that the interaction between porous β-TCP pellets and ibuprofen is weak. In vitro tests showed the complete release (100%) of ibuprofen due to Van der Waals forces [355]. Xiao and co-workers reported that an asymmetric coating formed from hydroxyapatite and gelatin on a Ti6Al4V alloy implant released ibuprofen for a minimum of 30 days. In addition, it was reported that in vitro studies in SBF led to the formation of apatite and the implant was fully covered after 14 days [356]. By comparison, Lin and co-workers released aspirin from a composite formed from PMMA and silica with various 3-(trimethoxysilyl) propyl methacrylate proportions and silica contents. It was observed that the release of the drug in PBS decreased with the increase in the 3-(trimethoxysilyl) propyl methacrylate content and increased with the silica content in the composites [357]. 

**Figure 37:**
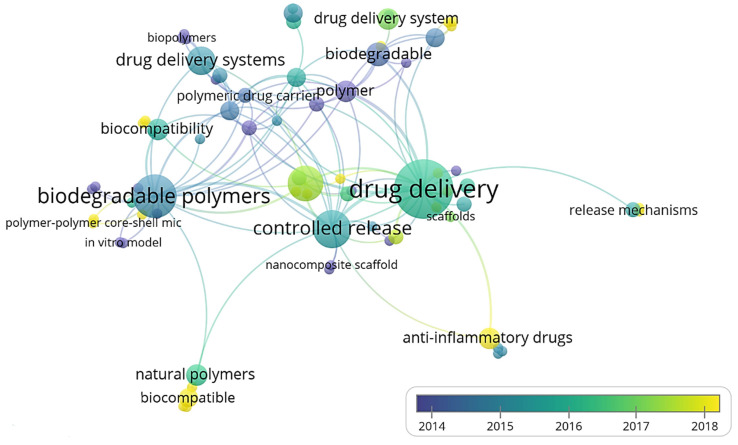
Scientometric bio-informatic mapping of anti-inflammatory drug delivery of biodegradable and natural polymeric biomaterials in hard tissue engineering.

Both non-enzymatic and enzymatic decomposition of biopolymers tend to produce an innocuous, functionalized biomimetic co-product [358,359,360]. Within the framework of bioactive novel genetic materials in specific targeted drug delivery applications, in particular, biopolymeric materials place a considerable accent on science, as illustrated in the bibliometric mapping analysis in Figure 37 [361,362,363,364,365,366,367,368]. Utilization with biocompatible polymers minimizes a drug’s adverse effects and negative consequences. Biopolymers, including biodegradable biopolymers, do not have a persistent inflammatory influence, and are characterized by high porosity and permeability, and outstanding therapeutic properties [369,370,371].

**Table 3:** Examples of systems used in hard tissue engineering [360].

Type	Fabrication Method	Materials		Applications
		Core	Shell	
Nanofiber	Co-axial electrospinning	PLGA	Collagen	Dual drug delivery systems for hard tissue engineering
	Co-axial electrospinning	PEO	PCL-PEG	Drug delivery systems for hard tissue engineering
	Co-axial electrospinning	PLLC	Collagen	Dual drug delivery systems for hard tissue engineering
Microfiber	Co-concentric extrusion	Tricalcium Phosphate and alginate	Alginate	Dual drug delivery systems for bone regeneration
Micropheres	Droplet coating	Alginate	Calcium silicate	Protein delivery control for hard tissue engineering
	Co-axial electrodropping	PLGA	Alginate	Dual drug delivery systems for hard tissue engineering (Dexamethasone and BMP2)
	Biomimetic approach	Gelatin	Calcium phosphate	Drug delivery systems for hard tissue engineering

**Table 4:** Examples of studies of drug delivery systems for osteomyelitis [337].

Class	Material	Antibiotic	Tested on Microorganism	Animal Model
Bioceramic	Calcium phosphate	Gentamicin	*S. aureus*	Rabbits
	Calcium sulphate	Moxifloxacin	Methicillin resistant *S. aureus*	Rabbits
	Hydroxyapatite	Vancomycin	*S. aureus*	Rabbits
Polymer	Collagen	Gentamicin	*S. aureus*	Rabbits
	PEG, PLGA	Tobramycin, Cefazolin	*S. aureus*	Rabbits
	Polylactide/polyglycolide	Gentamicin	*S. aureus*	Dogs
Bioactive glass	Borate	Vancomycin	Methicillin resistant *S. aureus*	Rabbits
	Boro-silicate	Ceftriaxone–sulbactam	*S. aureus*	Rabbits
Polymer composite	Chitosan, borate glass	Teicoplanin	*S. aureus*	Rabbits
	PLGA, bioactive glass	Ciprofloxacin	*S. aureus*	Rabbits

## 4. Concluding Remarks and Future Outlook

Biodegradable polymer nanocomposites and other natural polymeric biomaterials (which are usually a combination of two or more materials) possess distinctive characteristics fused with sufficient energy to ensure that the outcome benefits from the best properties of both materials. In contrast to an individual material, a polymer nanocomposite is composed of two materials, and thus combines two sets of properties. A polymer bio-nanocomposite is the combination of a drug with a natural- or bio-carrier using nanotechnology. The parameters of bio-nanocomposites are evaluated via their drug release profile determined in vivo and in vitro, and their bioavailability in a biological system. Bio-nanocomposites are a class of materials comprised of nanosized particles within a composition of other materials. Drug delivery systems represent an emerging area that is essential for the treatment of numerous diseases. These systems can be synthesized using various methods, depending on the applications for which they are required, such as anticancer or anti-inflammatory applications. Research is currently underway to develop controlled release systems loaded with natural products, such as medicinal plants or phenolic compounds, to treat different pathologies. It has been reported that the biggest challenge to the future development of nanotherapeutics is advancing the research on systems based on natural products that are capable of enabling a targeted release. Another research challenge is the design and testing of novel methods of controlling the interaction of nanomaterials with the body. This paper also emphasized that current methods aim to target the disadvantage of polymeric nanomaterials when applied to certain organs, such as the spleen and the liver. The employment of biodegradable and biorelated co-polymeric materials in the treatment of cancer, and particularly the utilization of these materials as processing methods and modes of delivery for efficacious anticancer medications, has played a pioneering role. The consolidation of insights from synthetic and biological domains indicates that a paradigm shift for the development of both biopolymeric drug and genetic delivery systems is required. Substantial technological breakthroughs relating to the fabrication of relatively new biopolymers, in addition to the comprehension of biological processes, have laid the path for this barrier to be overcome. Targeted polymeric drug delivery systems that rely on bacterial pathogens and viruses may have a virulent immunosuppressive effect on the body. In the near future, attempting to combine viewpoints from synthetic and biological areas will offer a novel framework for the development of biopolymeric targeted drug delivery applications.

## Figures and Tables

**Table 1:** Biodegradable polymers, with their structure and possible applications [110,112].

Biodegradable Polymers	Structure	Uses
Polylactic acid, poly(l-lactide), and poly(dl-lactide) family (PLA)	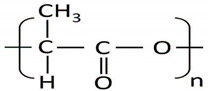	Sutures, prosthetics, drug delivery.
Polyglycolic acid (PGA)	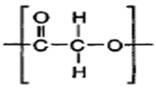	Drug carriers, sutures.
Polyl-lactide-*co*-glycolide family	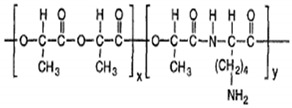	Drug delivery vehicle, sutures.
Poly(ortho esters)	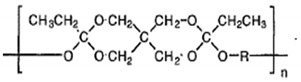	Ointments, drug delivery devices.
Polyanhydrides (e.g., polysebacic anhydride) (highly hydrophilic)	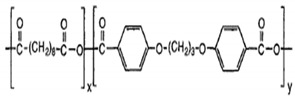	Drug delivery devices.
Polycarbonates	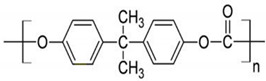	Drug delivery with some modifications, bone repair.
Phosphorus-containing polymers	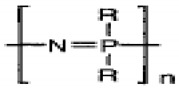	Implantable biomaterials.
Polydioxonone	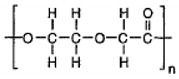	Plastic surgery, drug delivery, and tissue engineering.

**Table 2:** Water-soluble polymers [110].

Polymer	Structure	Uses
Saccharides (cellulose, dextran, chitin)	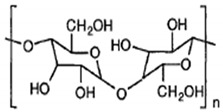	Cosmetics and health care products.
Acrylates and acrylamides (HEMA) PHEMA	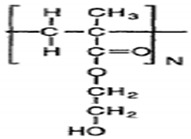	Contact lenses, catheters.
Polyethylene glycol (PEG)	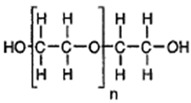	Drug delivery and as a stealth polymer.

## Data Availability

No data were used to support this study.
